# Supplementary material for: Mitochondrial dynamics regulate genome stability via control of caspase-dependent DNA damage
Source: Dev Cell. 2022 May 23;57(10):1211–1225.e6. doi: 10.1016/j.devcel.2022.03.019 (PMC9616799; doi:10.1016/j.devcel.2022.03.019)
Supplement: Document S1. Figures S1–S7 [file mmc1.pdf]

Developmental Cell, Volume *57*

Supplemental information

Mitochondrial dynamics regulate genome stability

via control of caspase-dependent DNA damage

Kai Cao, Joel S. Riley, Rosalie Heilig, Alfredo E. Montes-Gómez, Esmee Vringer, Kevin Berthenet, Catherine Cloix, Yassmin Elmasry, David G. Spiller, Gabriel Ichim, Kirsteen J. Campbell, Andrew P. Gilmore, and Stephen W.G. Tait

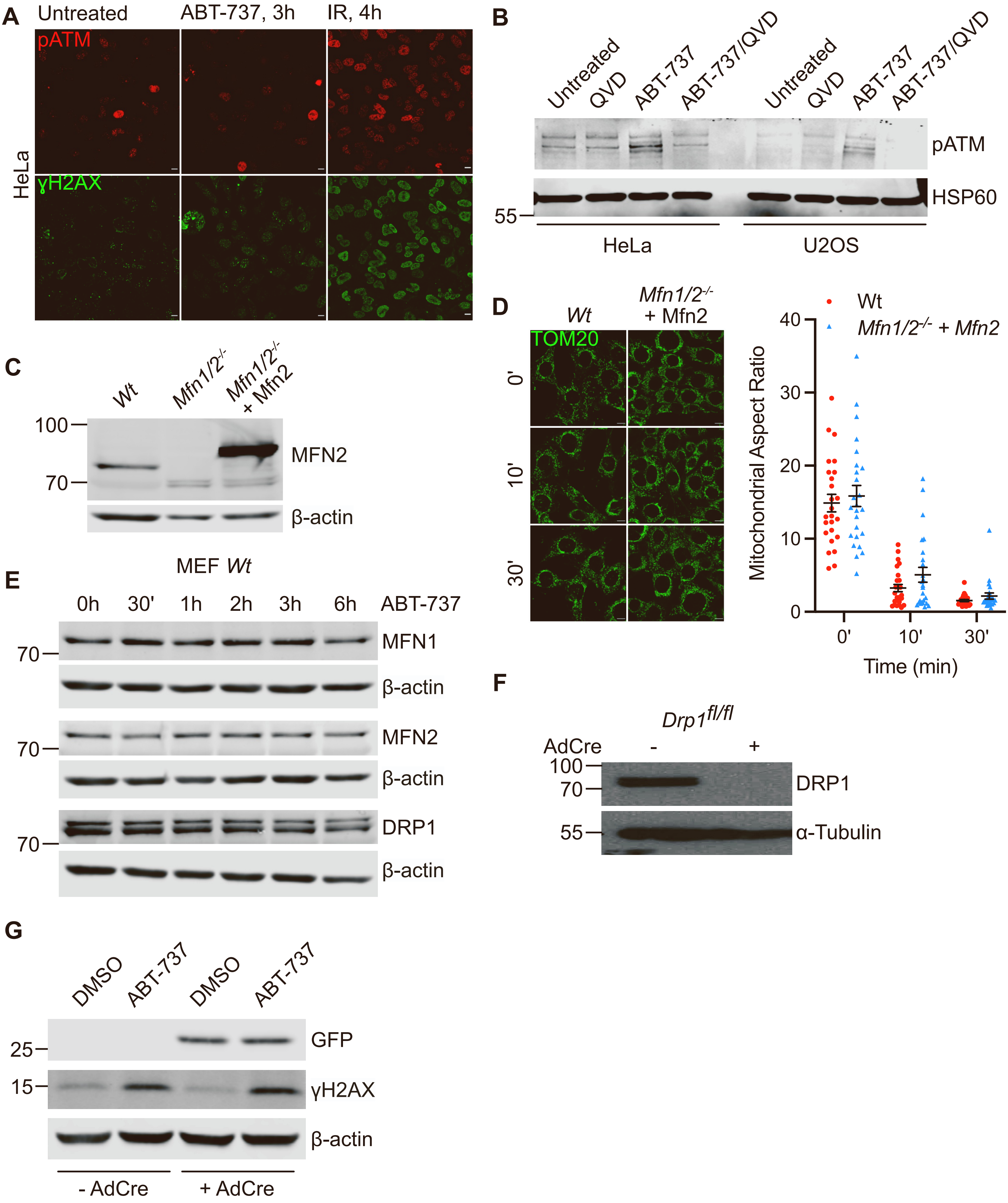
A
Untreated
ABT-737, 3h
IR, 4h
pATM
γH2AX
HeLa
B
Untreated
QVD
ABT-737
ABT-737/QVD
pATM
HSP60
55
HeLa
U2OS
C
Wt
Mfn1/2-/-
Mfn1/2-/- + Mfn2
100
70
MFN2
β-actin
D
Wt
Mfn1/2-/- + Mfn2
TOM20
0'
10'
30'
Wt
Mfn1/2-/- + Mfn2
Mitochondrial Aspect Ratio
40
30
20
10
0
Time (min)
E
MEF Wt
0h
30'
1h
2h
3h
6h
ABT-737
MFN1
β-actin
MFN2
DRP1
70
F
Drp1fl/fl
AdCre
-
+
100
70
55
DRP1
α-Tubulin
G
DMSO
ABT-737
GFP
γH2AX
β-actin
25
15
- AdCre
+ AdCre

Figure S1. Mitochondrial dynamics regulate DNA damage. Related to Figure 1

A) Airyscan images of HeLa cells treated with 10 μM ABT-737 for 3 h, or 20 Gy irradiation for 4 h. Cells were immunostained with anti-pATM (red) or anti-ɣH2AX (green) antibody. Images representative of 3 independent experiments. Scale bar = 10 μm

B) Western blot of HeLa and U2OS cells treated with 10 μM QVD, 10 μM ABT-737 or a combination of both for 3 h. Blots were immunoblotted for pATM and HSP60 and a representative of 2 independent experiments.

C) Western blot of *Wt*, *Mfn1/2*$^{-/-}$ and *Mfn1/2*$^{-/-}$ + Mfn2 MEF immunoblotted for MFN2 and β-actin. Representative of 2 independent experiments.

D) Airyscan images of *Wt* or *Mfn1/2*$^{-/-}$ + Mfn2 MEF treated with 10 μM CCCP for the indicated times and immunostained with anti-TOM20 antibody. Mitochondrial aspect ratio data is from 3 independent experiments. Data are expressed as mean ± SEM.

E) Western blot of *Wt* MEF treated with 10 μM ABT-737 for 3 h, and immunoblotted for MFN1, MFN2, DRP1 and β-actin. Data are representative of 3 independent experiments.

F) Western blot of *Drp1*$^{fl/fl}$ MEF with and without adenoviral Cre infection. Lysates immunoblotted for DRP1 and α-tubulin.

G) Western blot of loxP-STOP-loxP MEF infected or not with adenoviral Cre to induce YFP expression. Cells were treated with 10 μM ABT-737 for 3 h and YFP expression analysed by GFP immunoblotting, DNA damage by ɣH2AX and β-actin for loading control. Data is representative of 3 independent experiments.

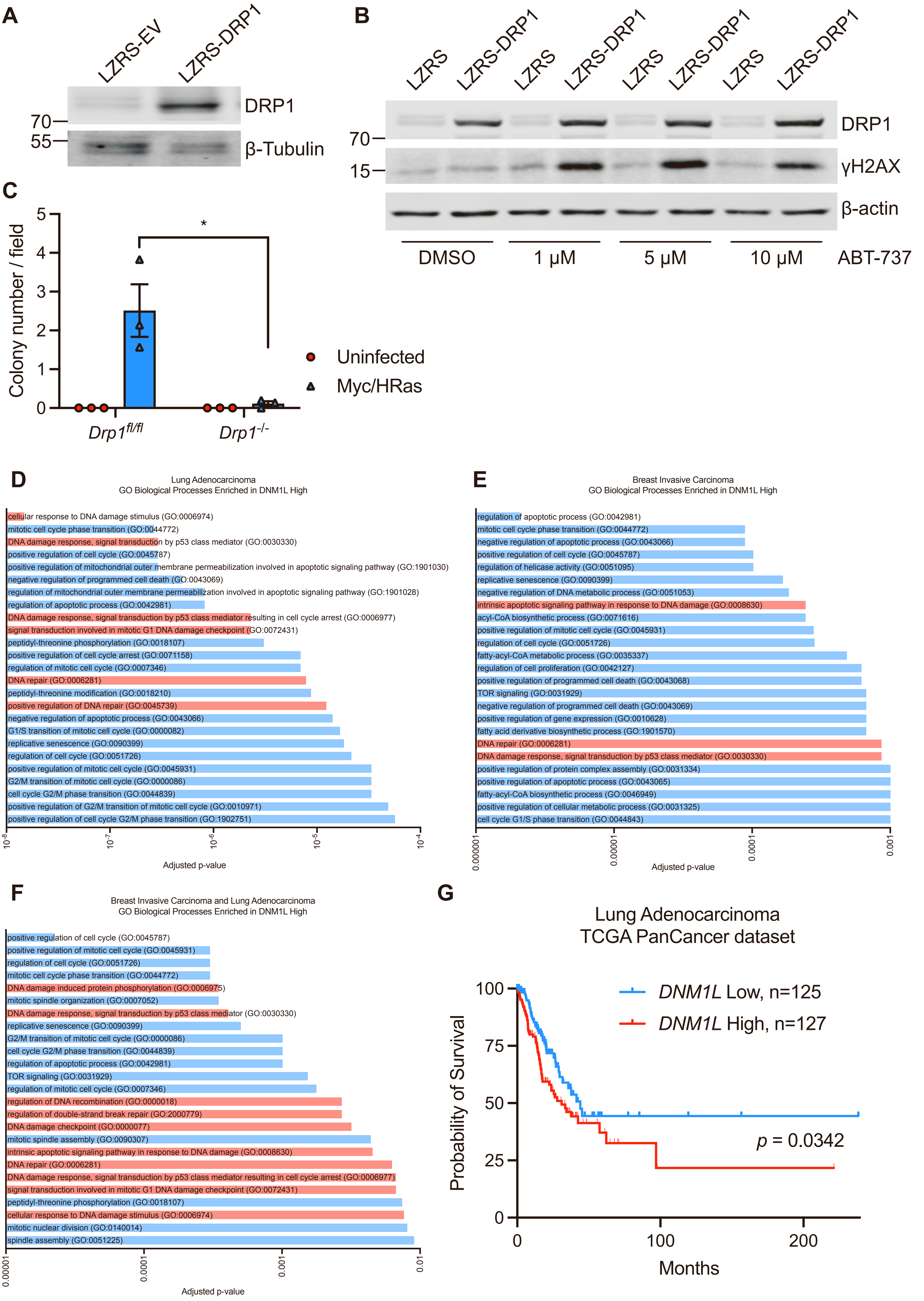

A
LZRS-EV
LZRS-DRP1
DRP1
70
55
β-Tubulin
B
LZRS
LZRS-DRP1
LZRS
LZRS-DRP1
LZRS
LZRS-DRP1
LZRS
LZRS-DRP1
DRP1
70
15
γH2AX
β-actin
DMSO
1 μM
5 μM
10 μM
ABT-737
C
Colony number / field
5
4
3
2
1
0
*
Drp1fl/fl
Drp1-/-
Uninfected
Myc/HRas
D
Lung Adenocarcinoma
GO Biological Processes Enriched in DNM1L High
cellular response to DNA damage stimulus (GO:0006974)
mitotic cell cycle phase transition (GO:0044772)
DNA damage response, signal transduction by p53 class mediator (GO:0030330)
positive regulation of cell cycle (GO:0045787)
positive regulation of mitochondrial outer membrane permeabilization involved in apoptotic signaling pathway (GO:1901030)
negative regulation of programmed cell death (GO:0043069)
regulation of mitochondrial outer membrane permeabilization involved in apoptotic signaling pathway (GO:1901028)
regulation of apoptotic process (GO:0042981)
DNA damage response, signal transduction by p53 class mediator resulting in cell cycle arrest (GO:0006977)
signal transduction involved in mitotic G1 DNA damage checkpoint (GO:0072431)
peptidyl-threonine phosphorylation (GO:0018107)
positive regulation of cell cycle arrest (GO:0071158)
regulation of mitotic cell cycle (GO:0007346)
DNA repair (GO:0006281)
peptidyl-threonine modification (GO:0018210)
positive regulation of DNA repair (GO:0045739)
negative regulation of apoptotic process (GO:0043066)
G1/S transition of mitotic cell cycle (GO:0000082)
replicative senescence (GO:0090399)
regulation of cell cycle (GO:0051726)
positive regulation of mitotic cell cycle (GO:0045931)
G2/M transition of mitotic cell cycle (GO:0000086)
cell cycle G2/M phase transition (GO:0044839)
positive regulation of G2/M transition of mitotic cell cycle (GO:0010971)
positive regulation of cell cycle G2/M phase transition (GO:1902751)
10-8
10-7
10-6
10-5
10-4
Adjusted p-value
E
Breast Invasive Carcinoma
GO Biological Processes Enriched in DNM1L High
regulation of apoptotic process (GO:0042981)
mitotic cell cycle phase transition (GO:0044772)
negative regulation of apoptotic process (GO:0043066)
positive regulation of cell cycle (GO:0045787)
regulation of helicase activity (GO:0051095)
replicative senescence (GO:0090399)
negative regulation of DNA metabolic process (GO:0051053)
intrinsic apoptotic signaling pathway in response to DNA damage (GO:0008630)
acyl-CoA biosynthetic process (GO:0071616)
positive regulation of mitotic cell cycle (GO:0045931)
regulation of cell cycle (GO:0051726)
fatty-acyl-CoA metabolic process (GO:0035337)
regulation of cell proliferation (GO:0042127)
positive regulation of programmed cell death (GO:0043068)
TOR signaling (GO:0031929)
negative regulation of programmed cell death (GO:0043069)
positive regulation of gene expression (GO:0010628)
fatty acid derivative biosynthetic process (GO:1901570)
DNA repair (GO:0006281)
DNA damage response, signal transduction by p53 class mediator (GO:0030330)
positive regulation of protein complex assembly (GO:0031334)
positive regulation of apoptotic process (GO:0043065)
fatty-acyl-CoA biosynthetic process (GO:0046949)
positive regulation of cellular metabolic process (GO:0031325)
cell cycle G1/S phase transition (GO:0044843)
0.000001
0.00001
0.0001
0.001
Adjusted p-value
F
Breast Invasive Carcinoma and Lung Adenocarcinoma
GO Biological Processes Enriched in DNM1L High
positive regulation of cell cycle (GO:0045787)
positive regulation of mitotic cell cycle (GO:0045931)
regulation of cell cycle (GO:0051726)
mitotic cell cycle phase transition (GO:0044772)
DNA damage induced protein phosphorylation (GO:0006975)
mitotic spindle organization (GO:0007052)
DNA damage response, signal transduction by p53 class mediator (GO:0030330)
replicative senescence (GO:0090399)
G2/M transition of mitotic cell cycle (GO:0000086)
cell cycle G2/M phase transition (GO:0044839)
regulation of apoptotic process (GO:0042981)
TOR signaling (GO:0031929)
regulation of mitotic cell cycle (GO:0007346)
regulation of DNA recombination (GO:0000018)
regulation of double-strand break repair (GO:2000779)
DNA damage checkpoint (GO:0000077)
mitotic spindle assembly (GO:0090307)
intrinsic apoptotic signaling pathway in response to DNA damage (GO:0008630)
DNA repair (GO:0006281)
DNA damage response, signal transduction by p53 class mediator resulting in cell cycle arrest (GO:0006977)
signal transduction involved in mitotic G1 DNA damage checkpoint (GO:0072431)
peptidyl-threonine phosphorylation (GO:0018107)
cellular response to DNA damage stimulus (GO:0006974)
mitotic nuclear division (GO:0140014)
spindle assembly (GO:0051225)
0.00001
0.0001
0.001
0.01
Adjusted p-value
G
Lung Adenocarcinoma
TCGA PanCancer dataset
DNM1L Low, n=125
DNM1L High, n=127
Probability of Survival
100
75
50
25
0
p = 0.0342
0
100
200
Months

Figure S2. Mitochondrial fission regulates DNA damage, transformation and cancer survival. Related to Figure 2

A) Western blot of MEF expressing LZRS empty control, or LZRS-DRP1. Lysates immunoblotted for DRP1 and β-tubulin.

B) MEF stably overexpressing LZRS-DRP1 or LZRS empty vector were treated with the indicated dose of ABT-737 for 3 h and lysates immunoblotted for DRP1, ɣH2AX and β-actin. Data are representative of 3 independent experiments.

C) *Drp1*$^{fl/fl}$ and *Drp1*$^{-/-}$ MEF infected with viruses encoding Myc and HRas were cultured in soft agar and colony number quantified. Data are from 3 independent experiments, expressed as mean ± SEM and analysed using student's t-test.

D) Gene ontology (GO) biological processes significantly up-regulated in DNM1L high expressing lung adenocarcinoma samples. Bars in red represent GO biological processes related to DNA damage and are expressed as adjusted p-values.

E) Gene ontology (GO) biological processes significantly up-regulated in DNM1L high expressing breast invasive carcinoma samples. Bars in red represent GO biological processes related to DNA damage and are expressed as adjusted p-values.

F) Gene ontology (GO) biological processes significantly up-regulated in DNM1L high expressing lung adenocarcinoma and breast invasive carcinoma samples. Bars in red represent GO biological processes related to DNA damage and are expressed as adjusted p-values.

G) Survival of lung adenocarcinoma patients. DNM1L Low refers to those patients in the lowest quartile of DNM1L mRNA expression, and DNM1L High refers to patients in the highest quartile. Significance is calculated by log-rank (Mantel-Cox) test.

Statistics: * $p \leq 0.05$, ** $p \leq 0.01$, *** $p \leq 0.001$

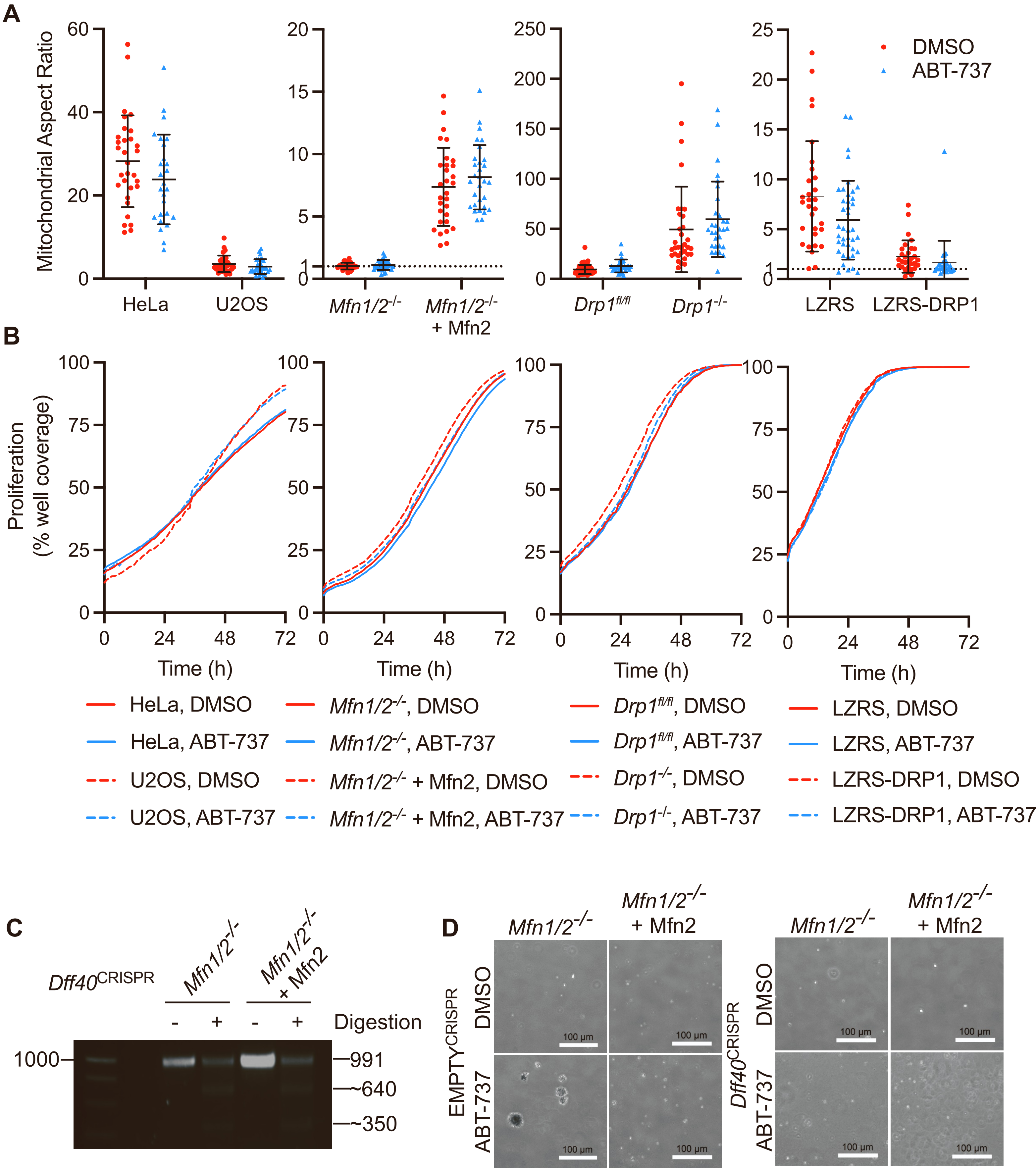
A
Mitochondrial Aspect Ratio
HeLa
U2OS
Mfn1/2-/-
Mfn1/2-/- + Mfn2
Drp1fl/fl
Drp1-/-
LZRS
LZRS-DRP1
DMSO
ABT-737
B
Proliferation (% well coverage)
Time (h)
HeLa, DMSO
HeLa, ABT-737
U2OS, DMSO
U2OS, ABT-737
Mfn1/2-/-, DMSO
Mfn1/2-/-, ABT-737
Mfn1/2-/- + Mfn2, DMSO
Mfn1/2-/- + Mfn2, ABT-737
Drp1fl/fl, DMSO
Drp1fl/fl, ABT-737
Drp1-/-, DMSO
Drp1-/-, ABT-737
LZRS, DMSO
LZRS, ABT-737
LZRS-DRP1, DMSO
LZRS-DRP1, ABT-737
C
Dff40CRISPR
Mfn1/2-/-
Mfn1/2-/- + Mfn2
Digestion
- + - +
1000
991
~640
~350
D
Mfn1/2-/-
Mfn1/2-/- + Mfn2
EMPTYCRISPR
Dff40CRISPR
DMSO
ABT-737
100 μm

Figure S3. Impact of mitochondrial fusion and fission machinery modulation on mitochondrial morphology, growth and CAD-dependent transformation. Related to Figure 4

A) Mitochondria aspect ratio of indicated cell lines treated with or without 10 µM ABT-737 for 3 h. Cells were immunostained for TOM20 and imaged using Airyscan microscopy. Mitochondrial aspect ratio was calculated from 3 independent experiments and expressed as mean (SD).
B) Proliferation of indicated cell lines treated with or without 10 µM ABT-737 for 3h, after which time the media was changed to media not containing ABT-737. Cells were imaged for 72 h.
C) T7 endonuclease I mismatch assay to assay for CAD/*Dff40* deletion.
D) Representative images of anchorage-independent growth of *Mfn1/2*$^{-/-}$ and *Mfn1*$^{-/-}$ MEF with and without CRISPR-Cas9-mediated *Dff40* deletion. Cells were passaged twenty times in 10 µM ABT-737. Scale bar = 100 µm. Data are quantified in **Figure 2K**.

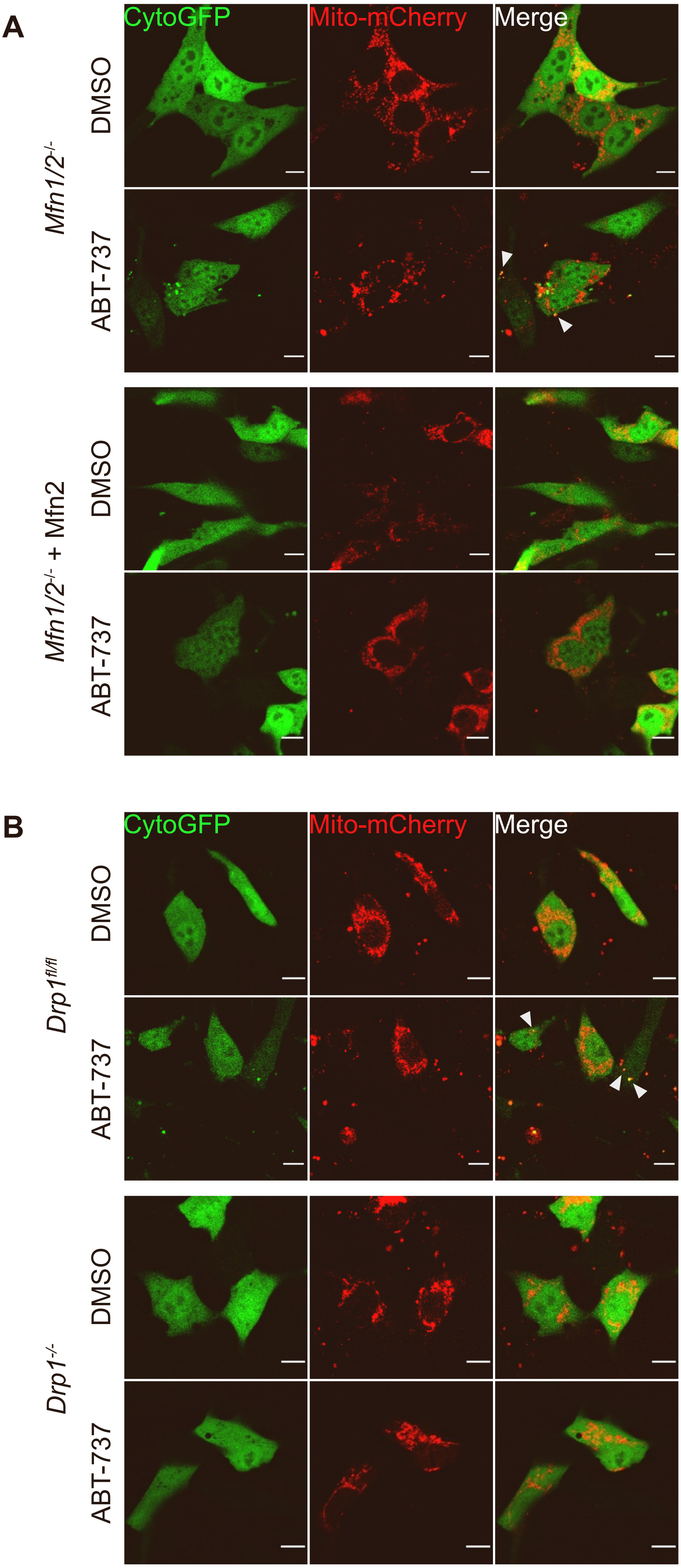

A
CytoGFP
Mito-mCherry
Merge
Mfn1/2-/-
DMSO
ABT-737
Mfn1/2-/- + Mfn2
DMSO
ABT-737
B
CytoGFP
Mito-mCherry
Merge
Drp1fl/fl
DMSO
ABT-737
Drp1-/-
DMSO
ABT-737

Figure S4. Visualisation of minority MOMP in cells with modulated mitochondrial dynamics. Related to Figure 5.

A) Confocal images of *Mfn1/2*$^{-/-}$ and *Mfn1/2*$^{-/-}$ + Mfn2 cells transiently expressing cytoGFP and mito-mCherry. Cells were treated with dimeriser and treated or not with 10 μM ABT-737 for 3 h before fixation, imaging and quantification of minority MOMP. Images are quantified in **Figure 3E**.

B) Confocal images of *Drp1*$^{fl/fl}$ and *Drp1*$^{-/-}$ cells transiently expressing cytoGFP and mito-mCherry. Cells were treated with dimeriser and treated or not with 10 μM ABT-737 for 3 h before fixation, imaging and quantification of minority MOMP. Images are quantified in **Figure 3F**.

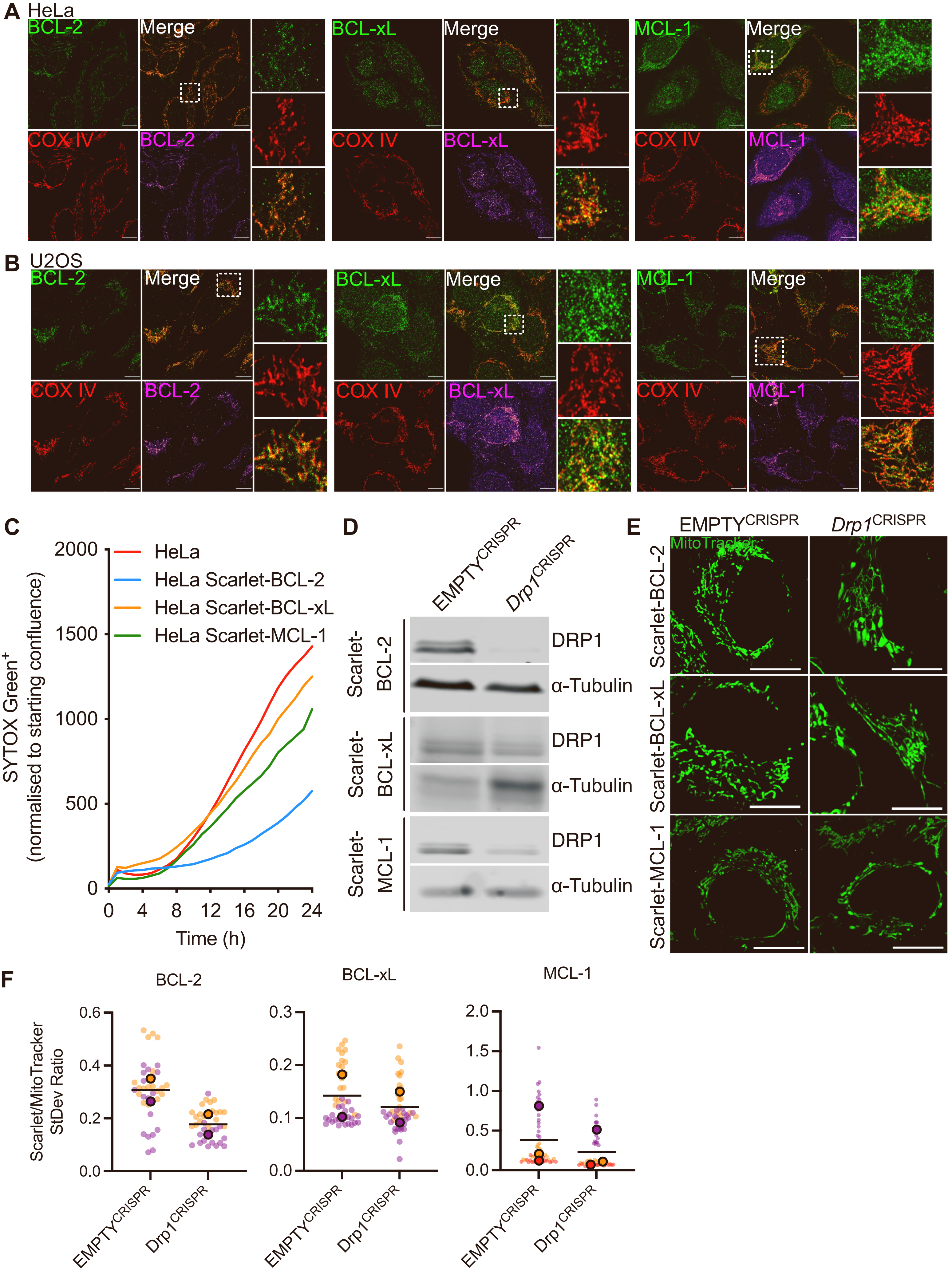
A HeLa
BCL-2
Merge
COX IV
BCL-2
BCL-xL
Merge
COX IV
BCL-xL
MCL-1
Merge
COX IV
MCL-1
B U2OS
BCL-2
Merge
COX IV
BCL-2
BCL-xL
Merge
COX IV
BCL-xL
MCL-1
Merge
COX IV
MCL-1
C
HeLa
HeLa Scarlet-BCL-2
HeLa Scarlet-BCL-xL
HeLa Scarlet-MCL-1
SYTOX Green+ (normalised to starting confluence)
Time (h)
D
EMPTYCRISPR
Drp1CRISPR
Scarlet-BCL-2
Scarlet-BCL-xL
Scarlet-MCL-1
DRP1
α-Tubulin
E
EMPTYCRISPR
Drp1CRISPR
MitoTracker
Scarlet-BCL-2
Scarlet-BCL-xL
Scarlet-MCL-1
F
BCL-2
BCL-xL
MCL-1
Scarlet/MitoTracker StDev Ratio
EMPTYCRISPR
Drp1CRISPR

Figure S5. Characterisation of BCL-2 family knockin cells. Related to Figure 6.

A) Airyscan images of HeLa cells immunostained for BCL-2, BCL-xL or MCL-1 (green) in combination with COX IV (red). Also shown are BCL-2, BCL-xL or MCL-1 images with a magenta LUT applied to visualise heterogeneity. Scale bar = 10 µm. Data are representative of 3 independent experiments.

B) Airyscan images of U2OS cells immunostained for BCL-2, BCL-xL or MCL-1 (green) in combination with COX IV (red). Also shown are BCL-2, BCL-xL or MCL-1 images with a magenta LUT applied to visualise heterogeneity. Scale bar = 10 µm. Data are representative of 3 independent experiments.

C) Live-cell IncuCyte analysis of HeLa and HeLa Scarlet-MCL-1 knockin cells treated with 10 µM ABT-737 and 2 µM S63845. Cells were incubated with SYTOX Green and SYTOX Green positivity was measured over time and normalised to starting confluency.

D) Western blots of HeLa Scarlet-BCL-2, BCL-xL and MCL-1 knockin cells with CRISPR-Cas9-mediated DRP1 deletion. Lysates immunoblotted for DRP1 and α-tubulin as a loading control.

E) Representative Airyscan images of mitochondrial structure in HeLa Scarlet-BCL-2, BCL-xL and MCL-1 knockin cells with CRISPR-Cas9-mediated DRP1 deletion. Scale bar = 10 µm. Data are representative of at least 2 independent experiments.

F) Quantification of standard deviation of Scarlet to MitoTracker signal in HeLa Scarlet BCL-2, BCL-xL and MCL-1 cells, with or without CRISPR-Cas9-mediated DRP1 deletion. Data are expressed as borderless points for individual cells and points with borders represent summary data for n = 2 biological replicates for BCL-2 and BCL-xL, and n = 3 for MCL-1. The line represents the mean.

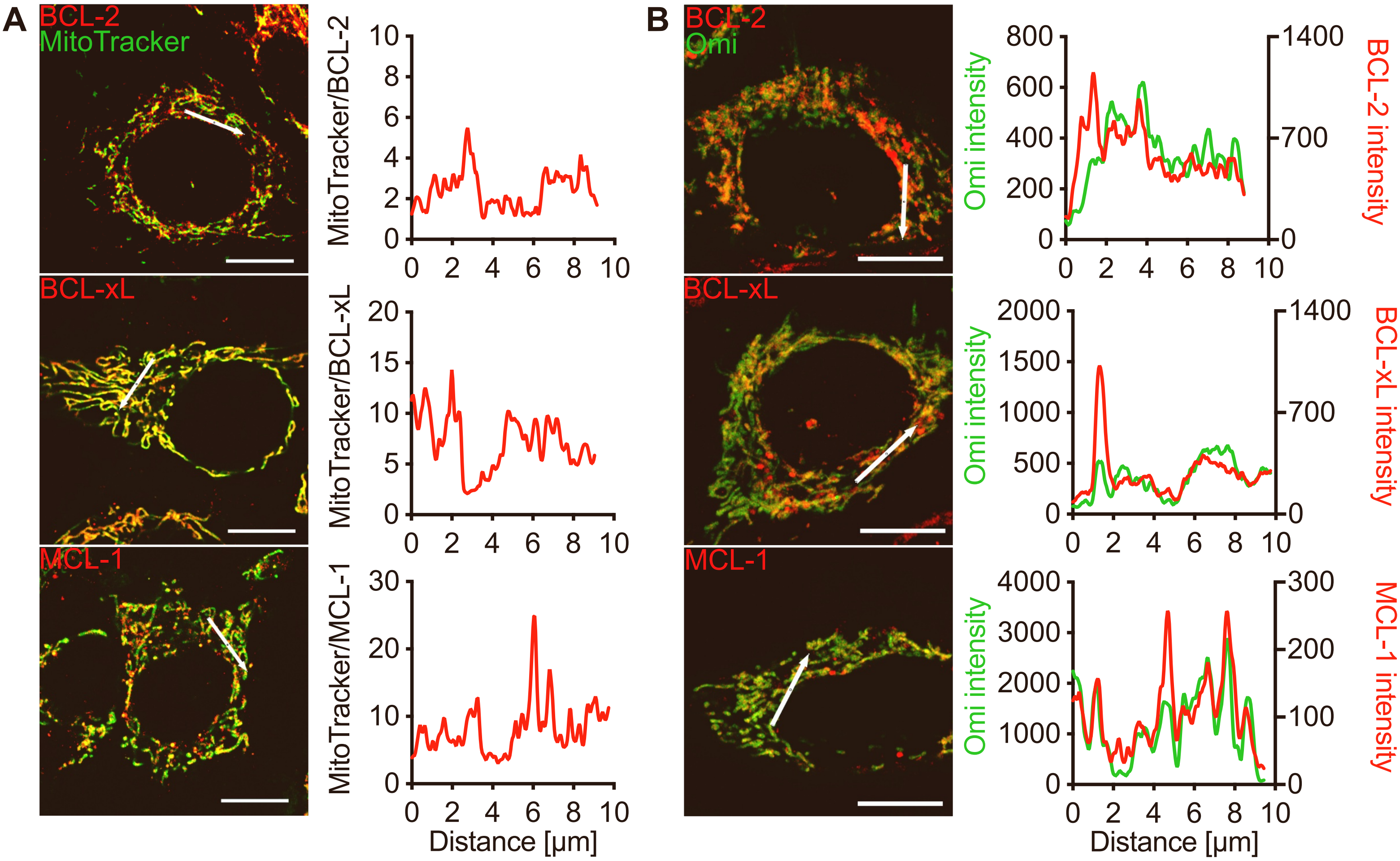

A
BCL-2
MitoTracker
BCL-xL
MCL-1
MitoTracker/BCL-2
MitoTracker/BCL-xL
MitoTracker/MCL-1
Distance [μm]
B
BCL-2
Omi
BCL-xL
MCL-1
Omi intensity
BCL-2 intensity
BCL-xL intensity
MCL-1 intensity
Distance [μm]

Figure S6. Mitochondrial localisation of BCL2-family knockins. Related to Figure 5.

A) Airyscan images of HeLa Scarlet-BCL-2, BCL-xL and MCL-1 knockin cells and MitoTracker. Line scans show ratio of MitoTracker to Scarlet-BCL-2. Scale bar = 10 μm. Data representative of 3 independent experiments.

B) Airyscan images of HeLa Scarlet-BCL-2, BCL-xL and MCL-1 knockin cells and Omi-GFP. Line scans show ratio of Omi-GFP intensity (green) and Scarlet-BCL-2 intensity (red). Scale bar = 10 μm. Data representative of 3 independent experiments.

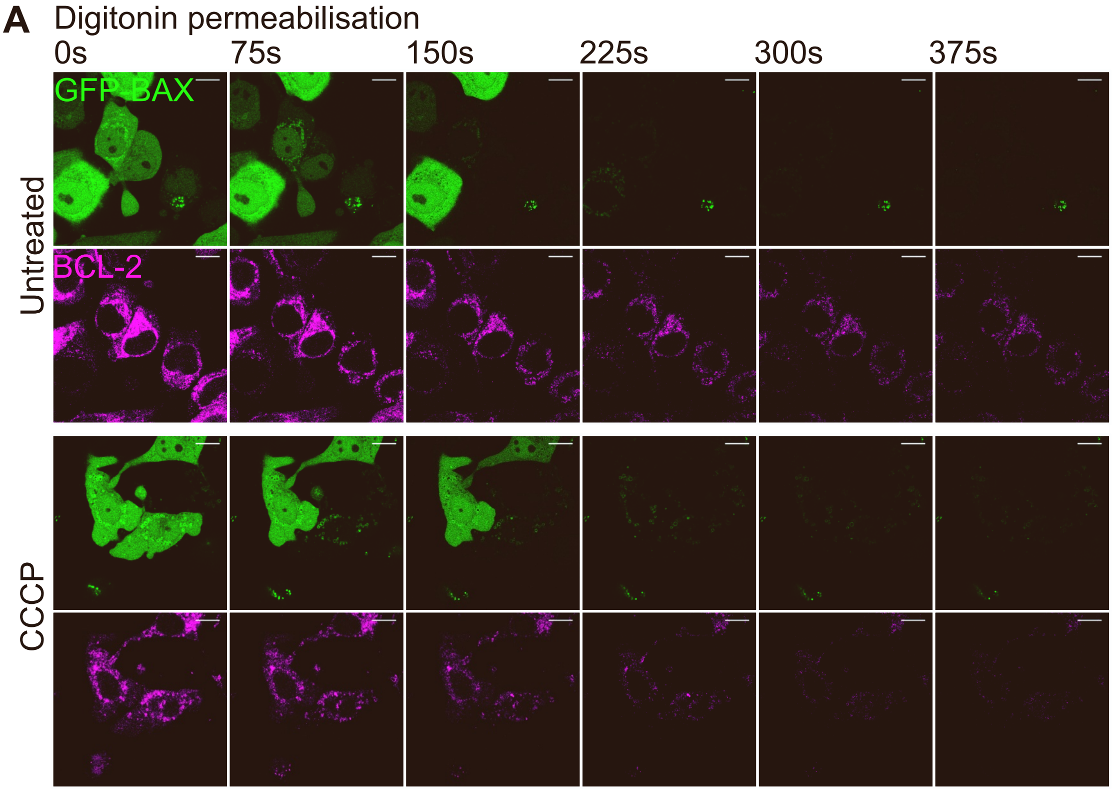
A
Digitonin permeabilisation
0s
75s
150s
225s
300s
375s
GFP-BAX
BCL-2
Untreated
CCCP

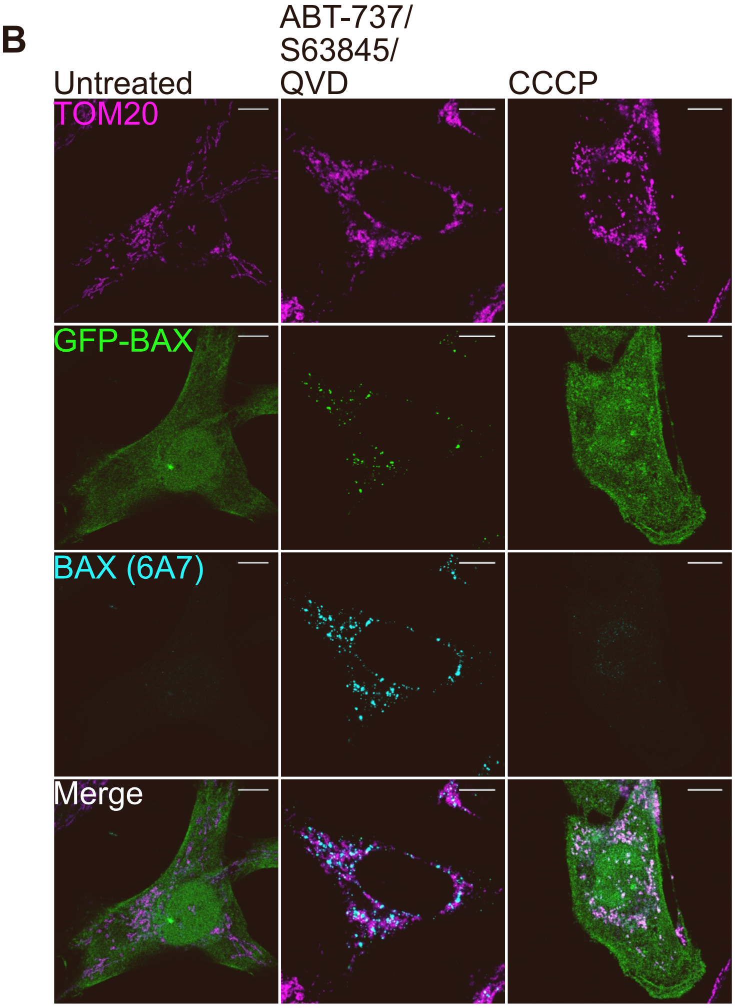
B
Untreated
ABT-737/
S63845/
QVD
CCCP
TOM20
GFP-BAX
BAX (6A7)
Merge

Figure S7. Mitochondrial dysfunction causes BAX accumulation on the mitochondrial outer membrane, but not BAX pore formation. Related to Figure 6.

A) Images of HeLa Scarlet-BCL-2 knockin (magenta) cells stably overexpressing GFP-BAX (green) treated 10 µM CCCP for 30 min before digitonin permeabilisation. Scale bar = 10 µm. See also **Videos S4, S5**.

B) Airyscan images of fixed HeLa cells stably overexpressing GFP-BAX (green) and treated with 10 µM CCCP or 10 µM ABT-737, 2 µM S63845 and 10 µM QVD for 3 h. Cells were immunostained with anti-TOM20 (magenta) and anti-BAX 6A7 (cyan). Scale bar = 10 µm. Images representative of 3 independent experiments.
