# Supplementary material for: Mitochondrial dynamics regulate genome stability via control of caspase-dependent DNA damage
Source: Dev Cell. 2022 May 23;57(10):1211–1225.e6. doi: 10.1016/j.devcel.2022.03.019 (PMC9616799; doi:10.1016/j.devcel.2022.03.019)
Supplement: Document S2. Article plus supplemental information [file mmc10.pdf]

Developmental Cell

Article

Mitochondrial dynamics regulate genome stability via control of caspase-dependent DNA damage

Graphical abstract

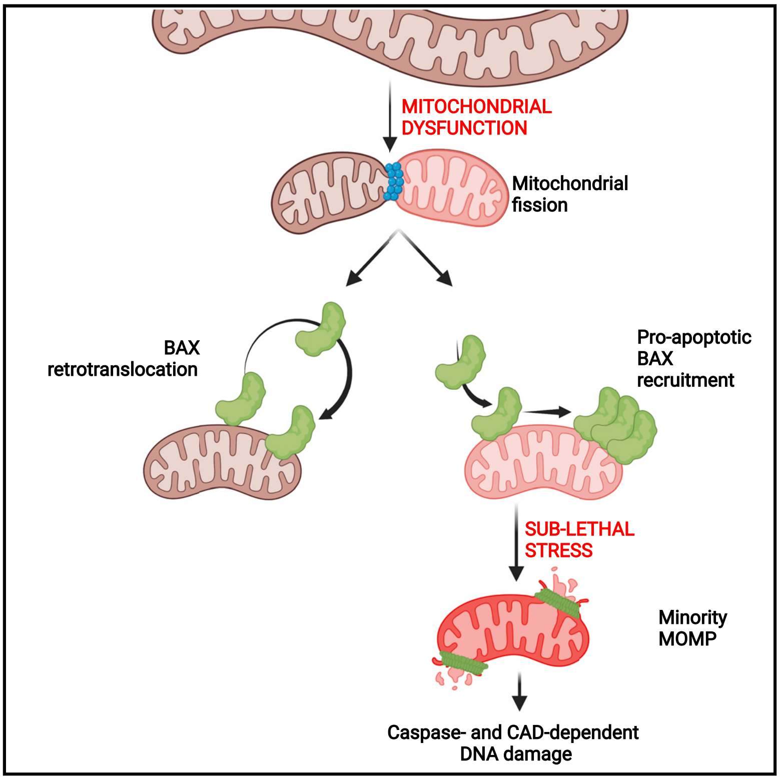

Highlights

- Mitochondrial fission caused by mitochondrial dysfunction promotes DNA damage
- Minority MOMP occurs on dysfunctional, fragmented mitochondria
- Inter-mitochondrial heterogeneity in apoptotic priming underpins minority MOMP
- BAX accumulates on dysfunctional, fragmented mitochondria, sensitizing them to MOMP

Authors

Kai Cao, Joel S. Riley, Rosalie Heilig, ..., Kirsteen J. Campbell, Andrew P. Gilmore, Stephen W.G. Tait

Correspondence

stephen.tait@glasgow.ac.uk

In brief

Sub-lethal apoptotic stress can cause oncogenic DNA damage. Investigating its basis, Cao, Riley, and colleagues report that mitochondrial dysfunction promotes sub-lethal apoptotic stress. Mitochondrial dysfunction causes permeabilization of select mitochondria through mitochondrial fission and pro-apoptotic BAX mitochondrial accumulation. Therefore, mitochondrial function and genome stability are interconnected via sub-lethal apoptotic stress.

Cao et al., 2022, Developmental Cell *57*, 1211–1225
May 23, 2022 © 2022 The Author(s). Published by Elsevier Inc.
https://doi.org/10.1016/j.devcel.2022.03.019

Developmental Cell

CellPress
OPEN ACCESS

Article

Mitochondrial dynamics regulate genome stability via control of caspase-dependent DNA damage

Kai Cao,[1,2,3,9] Joel S. Riley,[1,2,4,9] Rosalie Heilig,[1,2] Alfredo E. Montes-Gómez,[1,2] Esmee Vringer,[1,2] Kevin Berthenet,[5,6] Catherine Cloix,[1,2] Yassmin Elmasry,[1,2] David G. Spiller,[7] Gabriel Ichim,[5,6] Kirsteen J. Campbell,[1,2] Andrew P. Gilmore,[8] and Stephen W.G. Tait[1,2,10,*]

[1]Cancer Research UK Beatson Institute, Glasgow G61 1BD, UK
[2]Institute of Cancer Sciences, College of Medical, Veterinary and Life Sciences, University of Glasgow, Glasgow G61 1QH, UK
[3]Department of Chemistry and Biology, Faculty of Environment and Life Science, Beijing University of Technology, Beijing 100124, People's Republic of China
[4]Institute of Developmental Immunology, Biocenter, Medical University of Innsbruck, Innsbruck, Austria
[5]Cancer Research Centre of Lyon (CRCL), INSERM 1052, CNRS 5286, Lyon, France
[6]Cancer Cell Death Laboratory, Part of LabEx DEVweCAN, Université de Lyon, Lyon, France
[7]Systems Microscopy, Faculty of Biology, Medicine and Health, University of Manchester, Manchester M13 9PT, UK
[8]Wellcome Centre for Cell-Matrix Research, Faculty of Biology, Medicine and Health, Manchester Academic Science Centre, University of Manchester, Manchester M13 9PT, UK
[9]These authors contributed equally
[10]Lead contact
*Correspondence: stephen.tait@glasgow.ac.uk
https://doi.org/10.1016/j.devcel.2022.03.019

SUMMARY

Mitochondrial dysfunction is interconnected with cancer. Nevertheless, how defective mitochondria promote cancer is poorly understood. We find that mitochondrial dysfunction promotes DNA damage under conditions of increased apoptotic priming. Underlying this process, we reveal a key role for mitochondrial dynamics in the regulation of DNA damage and genome instability. The ability of mitochondrial dynamics to regulate oncogenic DNA damage centers upon the control of minority mitochondrial outer membrane permeabilization (MOMP), a process that enables non-lethal caspase activation leading to DNA damage. Mitochondrial fusion suppresses minority MOMP and its associated DNA damage by enabling homogeneous mitochondrial expression of anti-apoptotic BCL-2 proteins. Finally, we find that mitochondrial dysfunction inhibits pro-apoptotic BAX retrotranslocation, causing BAX mitochondrial localization and thereby promoting minority MOMP. Unexpectedly, these data reveal oncogenic effects of mitochondrial dysfunction that are mediated via mitochondrial dynamics and caspase-dependent DNA damage.

INTRODUCTION

Mitochondrial dysfunction has a pleiotropic impact on cancer (Giampazolias and Tait, 2016). For instance, mitochondrial respiratory complex proteins and TCA enzymes bearing tumor associated mutations generate oncometabolites (Isaacs et al., 2005; Pollard et al., 2007; Sciacovelli et al., 2016; Selak et al., 2005). Moreover, loss-of-function mutations in mitochondrial DNA (mtDNA) are common in cancer and have been shown to accelerate tumorigenesis (Gorelick et al., 2021; Smith et al., 2020). Nonetheless, how dysfunctional mitochondria promote cancer largely remains an open question.

While the inhibition of mitochondrial apoptosis has well-established oncogenic effects, through increased apoptotic priming, tumor cells are often sensitized to cell-killing cancer therapies (Certo et al., 2006; Singh et al., 2019). Mitochondria regulate apoptosis via mitochondrial outer membrane permeabilization (MOMP) (Bock and Tait, 2020). This key event releases soluble mitochondrial intermembrane space proteins into the cytoplasm, notably cytochrome *c*, which activate caspases proteases, causing rapid cellular demise. Because it dictates cell fate, mitochondrial outer membrane integrity is tightly regulated by BCL-2 protein family members (Campbell and Tait, 2018).

MOMP is usually considered a lethal point-of-no-return due to its extensive nature, often occurring in all mitochondria and coupled to an invariable loss of mitochondrial function (Goldstein et al., 2000; Lartigue et al., 2009; Rehm et al., 2003). However, we have previously described conditions whereby MOMP can be heterogenous, permitting cell survival (Ichim et al., 2015; Tait et al., 2010). Following a sub-lethal stress, a limited mitochondrial cohort selectively permeabilizes, which we termed "minority MOMP" (Ichim et al., 2015). Strikingly, minority MOMP can engage sub-lethal caspase activity promoting DNA damage that is dependent upon caspase-activated DNAse (CAD) (Ichim et al., 2015). By causing DNA damage, minority MOMP may contribute to the paradoxical oncogenic effects of

This is an open access article under the CC BY license (http://creativecommons.org/licenses/by/4.0/).

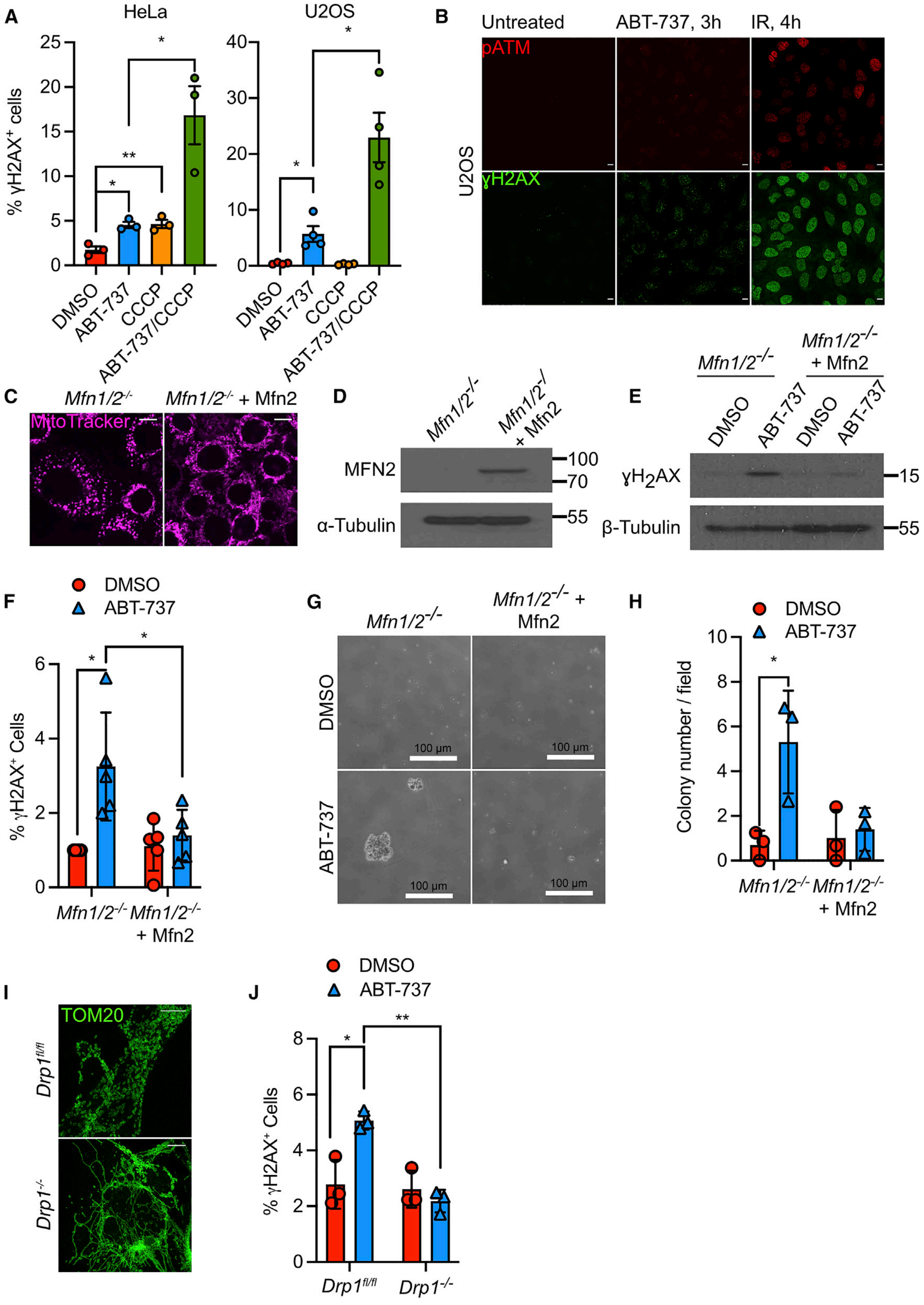

Figure 1. Mitochondrial dynamics regulate DNA damage

(A) Flow cytometric analysis of HeLa and U2OS cells treated with 10 μM CCCP for 30 min before treatment with 10 μM ABT-737 for 3 h. Cells were immunostained with anti-γH2AX antibody. Data represented as mean ± SEM from 3 independent experiments and analyzed using Student's t test.

(legend continued on next page)

apoptotic signaling reported in different studies (Ichim and Tait, 2016). Moreover, minority MOMP has been recently implicated in an expanding array of functions, including increased cancer aggressiveness, innate immunity, and inflammation triggered by mtDNA double-strand breaks (Berthenet et al., 2020; Brokatzky et al., 2019; Tigano et al., 2021).

Here, we investigated the relationship between mitochondrial dysfunction and DNA damage. Surprisingly, we uncovered a key role for mitochondrial dynamics in the regulation of DNA damage. Mitochondrial fission, a consequence of mitochondrial dysfunction, promotes minority MOMP, causing caspase-dependent DNA damage and genome instability. Secondly, we find reduced retrotranslocation of pro-apoptotic BAX on dysfunctional mitochondria, thus facilitating minority MOMP. These data reveal an unanticipated link between mitochondrial dysfunction and oncogenic DNA damage that is mediated through minority MOMP and caspase activity.

RESULTS

Mitochondrial dynamics regulate DNA damage

We aimed to understand how mitochondrial dysfunction can be oncogenic. Given the tumor-promoting roles of DNA damage, we initially investigated its interconnection with mitochondrial function. To cause mitochondrial dysfunction, U2OS and HeLa cells were treated with the uncoupler, carbonyl cyanide *m*-chlorophenyl hydrazone (CCCP). In order to phenocopy increased apoptotic priming that is found in pre-malignant and tumor cells, we co-treated cells with ABT-737, a BH3-mimetic compound that selectively neutralizes anti-apoptotic BCL-2, BCL-xL, and BCL-w (Oltersdorf et al., 2005). The response to DNA damage was measured by ɣH2AX staining and flow cytometry. In both HeLa and U2OS cells, BH3-mimetic treatment led to an increase in ɣH2AX-positive cells that was significantly enhanced by combined treatment with CCCP, consistent with mitochondrial dysfunction promoting DNA damage (Figure 1A). Given the low level of ɣH2AX-positive cells observed by flow cytometry, we visualized the degree of DNA damage in U2OS and HeLa cells treated with ABT-737 by indirect immunofluorescence and western blot. In agreement with the flow cytometry data, we observed a low but detectable level of ɣH2AX and pATM foci in cells treated with ABT-737 (Figure 1B; Figures S1A and S1B). Mitochondrial dynamics and function are tightly interconnected, such that mitochondrial dysfunction causes mitochondrial fission. We therefore investigated whether mitochondrial dynamics affected DNA damage triggered by BH3-mimetic treatment. To disrupt mitochondrial fusion, we used *Mfn1/2*$^{-/-}$ murine embryonic fibroblasts (*Mfn1/2*$^{-/-}$ MEFs) and, as control, reconstituted these cells with MFN2 (*Mfn1/2*$^{-/-}$ + MFN2 MEF). As expected, *Mfn1/2*$^{-/-}$ MEF displayed a hyper-fragmented mitochondrial network whereas MFN2 reconstitution of these cells (*Mfn1/2*$^{-/-}$ + MFN2) restored mitochondrial fusion, resulting in a filamentous mitochondrial network, but did not affect growth following treatment with ABT-737 (Figures 1C and 1D; Figures S3A and S3B). Of note, although MFN2 protein expression is higher in *Mfn1/2*$^{-/-}$ MEF reconstituted with MFN2 compared with wild-type (WT) MEF, mitochondrial fusion was not enhanced in *Mfn1/2*$^{-/-}$ + MFN2 MEF, and mitochondrial fragmentation occurred with the same kinetics when exposed to CCCP, relative to WT MEF (Figures S1C and S1D). Furthermore, expression of mitochondrial fusion proteins MFN1 and MFN2 and the mitochondrial fission protein DRP1 were not changed following ABT-737 treatment (Figure S1E). *Mfn1/2*$^{-/-}$ and *Mfn1/2*$^{-/-}$ + Mfn2 MEF were treated with ABT-737 (10 μM, 3 h) and the DNA damage response was assessed by analyzing ɣH2AX levels by western blot or by flow cytometry (Figures 1E and 1F). *Mfn1/2*$^{-/-}$ MEF exhibited increased ɣH2AX, consistent with mitochondrial fission promoting DNA damage. Because DNA damage can be oncogenic, we investigated whether cells with extensive mitochondrial fission were more prone to transformation. *Mfn1/2*$^{-/-}$ and *Mfn1/2*$^{-/-}$ + MFN2 MEF were passaged repeatedly in ABT-737. Following treatment, cells were assayed for transformation *in vitro* by determining anchorage-independent growth in soft agar. Specifically following culture in ABT-737, *Mfn1/2*$^{-/-}$ MEF formed colonies more readily than *Mfn1*$^{-/-}$ + MFN2 MEF (Figures 1G and 1H). MFN2 can have functions independent of mitochondrial fusion, for instance, mitochondrial-endoplasmic reticulum (ER) tethering (de Brito and Scorrano, 2008). Therefore, we investigated whether mitochondrial dynamics regulate DNA damage through an alternative approach by inhibiting mitochondrial fission. DRP1 plays a central role in mitochondrial fission (Ishihara et al., 2009; Wakabayashi et al., 2009). To inhibit mitochondrial fission, we used *Drp1*$^{fl/fl}$ MEF, which when infected with adenoviral Cre,

(B) Airyscan images of U2OS cells treated with 10 μM ABT-737 for 3 h or 20-Gy irradiation for 4 h. Cells were immunostained with anti-pATM (red) or anti-ɣH2AX (green) antibody. Images representative of 3 independent experiments. Scale bars, 10 μm.

(C) Airyscan images of *Mfn1/2*$^{-/-}$ and *Mfn1/2*$^{-/-}$ + Mfn2 MEF, immunostained with anti-TOM20 antibody. Scale bars, 10 μm.

(D) Immunoblot of MFN2 and β-tubulin (loading control) in *Mfn1/2*$^{-/-}$ and *Mfn1/2*$^{-/-}$ + Mfn2 MEF.

(E) Immunoblot of ɣH2AX and β-tubulin (loading control) in *Mfn1/2*$^{-/-}$ and *Mfn1*$^{-/-}$ + MFN2 MEF treated with 10 μM ABT-737 for 3 h. Data are representative of 3 independent experiments.

(F) Flow cytometric analysis of ɣH2AX expression in *Mfn1/2*$^{-/-}$ and *Mfn1/2*$^{-/-}$ + MFN2 MEF treated with 10 μM ABT-737 for 3 h. Data represented as mean (SD) from 5 independent experiments and analyzed using Student's t test.

(G) *Mfn1/2*$^{-/-}$ and *Mfn1/2*$^{-/-}$ + MFN2 MEF were cultured for twenty passages in 10 μM ABT-737 and their anchorage-independent growth assessed by soft agar assay. Representative images from 3 independent experiments shown.

(H) Quantification of anchorage-independent growth in soft agar from (G). Data are expressed as mean (SD) from 3 independent experiments and analyzed using Student's t test.

(I) Airyscan images of *Drp1*$^{fl/fl}$ MEF infected with AdCre (*Drp1*$^{-/-}$) and immunostained with anti-TOM20 antibody. Scale bars, 10 μm.

(J) Flow cytometric analysis of ɣH2AX expression in *Drp1*$^{fl/fl}$ and *Drp1*$^{-/-}$ MEF treated with 10 μM ABT-737 for 3 h. Data are expressed as mean ± SEM from 3 independent experiments and analyzed using Student's t test.

Statistics: *$p \leq 0.05$, **$p \leq 0.01$, ***$p \leq 0.001$.

See also Figure S1.

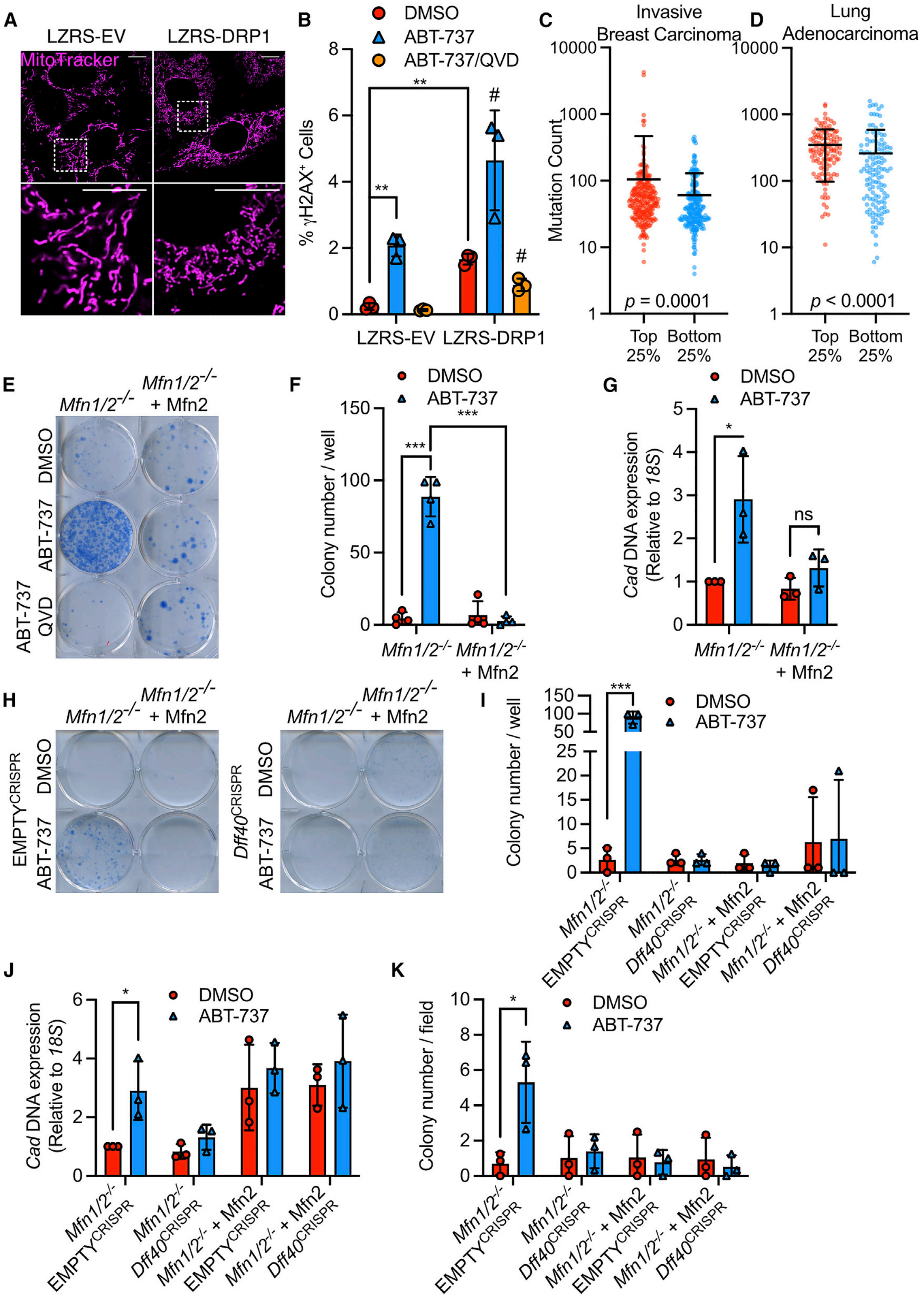

Figure 2. Mitochondrial dynamics regulate DNA damage and genome instability in a caspase- and CAD-dependent manner

(A) Airyscan images of MEF overexpressing LZRS-DRP1 or LZRS empty vector, stained with MitoTracker Deep Red. Lower panels represent the area in the region depicted. Scale bars, 10 μm.

(legend continued on next page)

efficiently delete Drp1, causing a hyperfused mitochondrial network (Figure 1I; Figures S1F and S3A). Infection with adenoviral Cre did not cause DNA damage nor impact ABT-737-induced DNA damage or growth following ABT-737 treatment (Figures S1G and S3B). *Drp1*$^{fl/fl}$ and *Drp1*$^{-/-}$ MEF were treated with ABT-737, and γH2AX was measured by flow cytometry, as before. MEF expressing DRP1 have elevated levels of γH2AX after exposure to ABT-737, but this was completely abolished in DRP1-deficient cells (Figure 1J). These data suggest that mitochondrial dysfunction and fission promote oncogenic DNA damage and transformation.

Mitochondrial dynamics regulate DNA damage and genome instability in a caspase- and CAD-dependent manner

We next sought to understand how mitochondrial dynamics regulate DNA damage. Because we had found that pro-apoptotic BH3-mimetic treatment potentiated DNA damage, we investigated a role for apoptotic caspase function. WT MEF or MEF overexpressing DRP1 were treated with the pan-caspase inhibitor quinolyl-valyl-O-methylaspartyl-[2,6-difluorophenoxy]-methyl ketone (qVD-OPh), and γH2AX was measured by flow cytometry and western blot. MEF cells overexpressing DRP1 displayed a more fragmented mitochondrial network and had higher levels of γH2AX compared with their empty vector counterparts, consistent with our earlier data, but showed similar proliferation rates following treatment with ABT-737 (Figures 2A and 2B; Figures S2A, S2B, S3A, and S3B). Crucially, γH2AX was prevented by treatment with the pan-caspase inhibitor qVD-OPh, demonstrating a key role for caspase activation in DNA damage (Figure 2B). We next set out to establish whether loss of DRP1 impacted oncogenic transformation following repeated culture in ABT-737; however, loss of DRP1 alone was sufficient to render cells resistant to Myc/Hras-induced transformation (Figure S2C), consistent with the results of Serasinghe and colleagues (Serasinghe et al., 2015). Given these findings, we investigated a possible correlation between the expression of the mitochondrial fission protein, DRP1, and mutational burden in cancer. TCGA PanCancer Atlas studies were investigated through cBioportal. Of these, a significant association between increased mutational count in *DNM1L* mRNA high quartile versus *DNM1L* mRNA low quartile was found in invasive breast carcinoma and lung adenocarcinoma (out of 22 studies), with the inverse relationship not observed in any cancer type (Figures 2C and 2D, and data not shown). In both invasive breast cancer and lung adenocarcinoma, DNA damage response pathways were enriched in the *DNM1L* mRNA high quartile, consistent with engagement of DNA damage (Figures S2D, S2E, and S2F; Table S1). Moreover, high *DNM1L* mRNA expression correlates with poorer survival in a cohort of lung adenocarcinoma (Figure S2G). To further investigate the role of caspase activity, we investigated the impact of mitochondrial dynamics upon genome instability. To this end, we used the N-phosphonoacetyl-L-aspartate (PALA) assay in which gene amplification of CAD (carbamyl phosphate synthetase/aspartate transcarbamylase/dihydro-orotase, note that this is distinct from CAD described later) enables resistance to PALA (Wahl et al., 1979). To determine whether alterations in mitochondrial dynamics also affect genome instability dependent upon caspase activity, we passaged *Mfn1/2*$^{-/-}$ and *Mfn1/2*$^{-/-}$ + MFN2 MEF with sub-lethal doses of ABT-737 in the presence or absence of qVD-OPh. Following treatment, cells were grown in the presence of PALA and clonogenic survival was measured (Figure 2E). Importantly, ABT-737-treated *Mfn1/2*$^{-/-}$ MEF gave significantly more colonies than *Mfn1/2*$^{-/-}$ + MFN2 following PALA treatment, in a caspase-dependent manner (Figures 2E and 2F). In line with increased survival following PALA treatment, qPCR revealed amplification of the *Cad* locus only in *Mfn1/2*$^{-/-}$ MEF repeatedly treated with ABT-737 (Figure 2G). We and others have previously found that non-lethal caspase activity can cause DNA damage and genome instability, dependent upon caspase-activated DNAse (CAD) (Ichim et al., 2015; Lovric and Hawkins, 2010). To examine the role of CAD in genomic instability we used the *Mfn1/2*$^{-/-}$ and *Mfn1/2*$^{-/-}$ + MFN2 MEF in which we deleted the *Dff40* gene (encoding CAD) using CRISPR-Cas9 genome editing (Figure S3C). As before, *Mfn1/2*$^{-/-}$ cells resisted PALA treatment and efficiently grew as colonies following ABT-737 treatment, whereas *Mfn1/2*$^{-/-}$ + MFN2 cells did not (Figures 2H and 2I). However, deletion of CAD completely abrogated clonogenic

(B) Flow cytometric analysis of MEF stably overexpressing LZRS control or LZRS-DRP1, treated with 10 μM ABT-737, with and without 20 μM qVD-OPh, for 3 h. Data are expressed at mean (SD) from 3 independent experiments and analyzed using Student's t test.
(C) Mutation counts in patient lung adenocarcinoma samples from the highest and lowest DNM1L mRNA quartiles. Significance is analyzed by Mann-Whitney test. Data points represent individual patient samples, bar represents mean (SD).
(D) Mutation counts in patient breast invasive carcinoma cancer samples from the highest and lowest DNM1L mRNA quartiles. Significance is analyzed by Mann-Whitney test. Data points represent individual patient samples, bar represents mean (SD).
(E) *Mfn1/2*$^{-/-}$ and *Mfn1/2*$^{-/-}$ + Mfn2 MEF were treated daily for twenty passages with 10 μM ABT-737, with and without 20 μM qVD-OPh. Clonogenic survival was performed in the presence of 100 μM PALA. Data are a representative example of 4 independent experiments.
(F) Quantification of clonogenic outgrowth from 4 independent experiments. Data are expressed as mean (SD) and analyzed using Student's t test.
(G) Quantification of *Cad* DNA levels in *Mfn1/2*$^{-/-}$ and *Mfn1/2*$^{-/-}$ + MFN2 MEF, treated with or without 10 μM ABT-737. Data are expressed as mean (SD) from 3 independent experiments and analyzed using Student's t test.
(H) *Mfn1/2*$^{-/-}$ and *Mfn1/2*$^{-/-}$ + MFN2 MEF, with and without CRISPR-Cas9-mediated *Dff40* deletion, treated daily for twenty passages with 10 μM ABT-737, with and without 20 μM QVD. Clonogenic survival was performed in the presence of 100 μM PALA. Data are a representative example of 3 independent experiments.
(I) Quantification of clonogenic outgrowth from (H) from 3 independent experiments. Data are expressed as mean (SD) and analyzed using Student's t test.
(J) Quantification of *Cad* DNA levels in *Mfn1/2*$^{-/-}$ and *Mfn1/2*$^{-/-}$ + MFN2 MEF, with and without *Dff40* deletion, and treated with or without 10 μM ABT-737. Data are expressed as mean (SD) from 3 independent experiments and analyzed using Student's t test.
(K) *Mfn1/2*$^{-/-}$ and *Mfn1/2*$^{-/-}$ + MFN2 MEF, with and without *Dff40* deletion, cultured for twenty passages in 10 μM ABT-737 and their anchorage-independent growth assessed by soft agar assay. Data are expressed as mean (SD) from 3 independent experiments and analyzed using Student's t test.
Statistics: *$p \leq 0.05$, **$p \leq 0.01$, ***$p \leq 0.001$.
See also Figures S2 and S3 and Table S1.

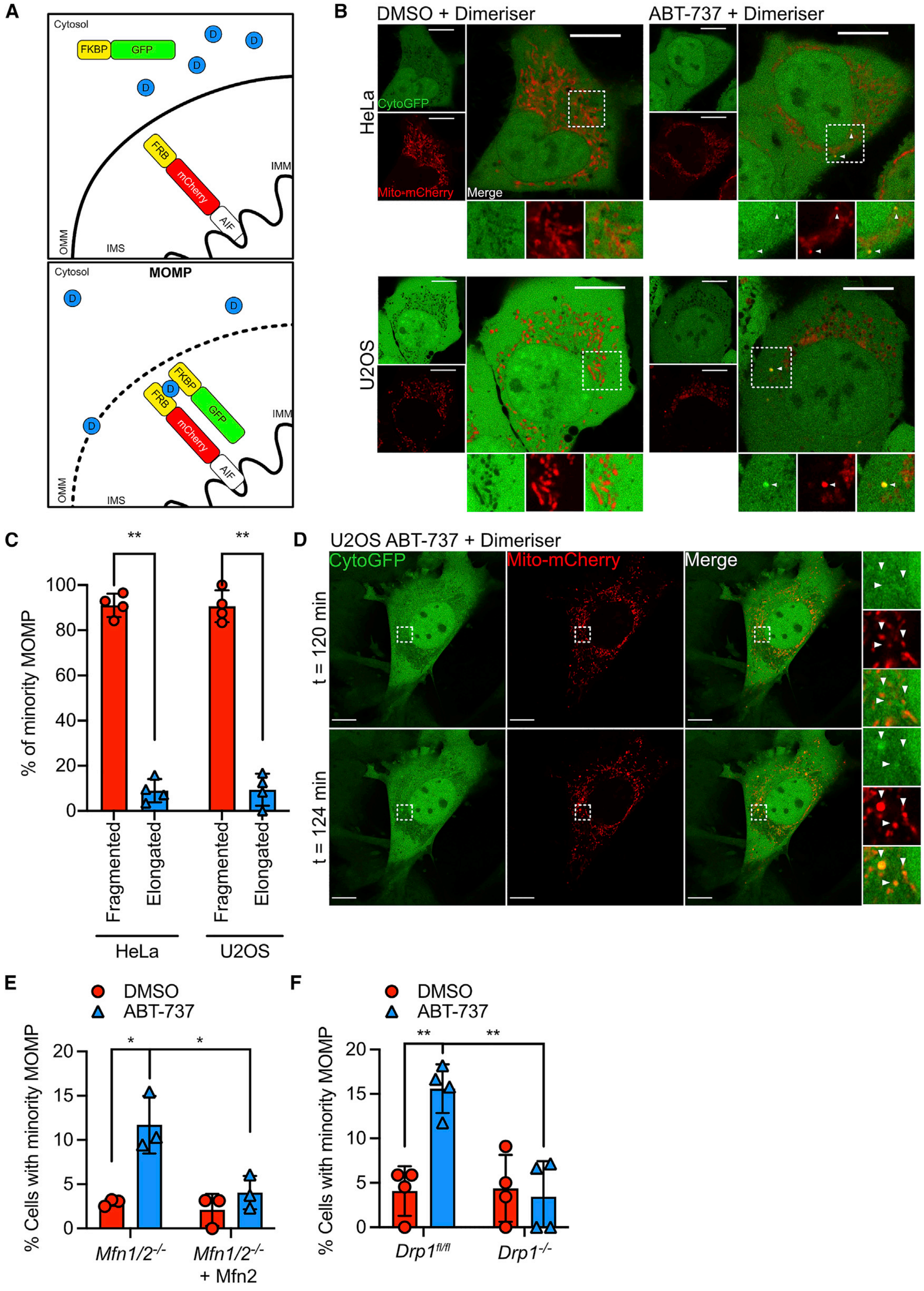

Figure 3. Minority MOMP occurs on fragmented mitochondria and is regulated by mitochondrial dynamics

(A) Schematic of the MOMP reporter.

(B) Fixed super-resolution Airyscan images of HeLa and U2OS cells transfected with cytoGFP (green) and mito-mCherry (red). Cells were treated with 10 μM ABT-737 for 3 h in the presence of dimerizer. Scale bars, 10 μm.

(legend continued on next page)

potential. *Cad* DNA expression and anchorage-independent growth were also diminished following ABT-737 treatment in CAD/*Dff40* deleted cells as compared with their controls (Figures 2J and 2K; Figure S3D). Together, these data show that mitochondrial fission promotes genome instability in a caspase- and CAD-dependent manner.

Minority MOMP occurs on fragmented mitochondria and is regulated by mitochondrial dynamics

We have previously found that permeabilization of a small number of mitochondria in a cell—called minority MOMP—can engage non-lethal caspase activity, causing CAD activation and DNA damage (Ichim et al., 2015). This knowledge, coupled with our previous data, led us to investigate a role for mitochondrial dynamics in the regulation of minority MOMP. To address this, we combined super-resolution Airyscan confocal microscopy together with our fluorescent reporter that allows detection of minority MOMP (Ichim et al., 2015). This reporter comprises cytosolic FKBP-GFP (cytoGFP) and mitochondrial inner membrane targeted FRB-mCherry (mito-mCherry). Upon loss of mitochondrial outer membrane integrity, and in the presence of chemical heterodimerizer (AP21967), these two proteins bind to one another, recruiting cytoGFP to the permeabilized mitochondria (Figure 3A). HeLa or U2OS were treated with a non-lethal dose of BH3-mimetic ABT-737 (10 μM) for 3 h. Consistent with our previous data, this treatment was sufficient to engage minority MOMP, as evidenced by localization of cytoGFP to specific mitochondria (Figure 3B). Super-resolution analysis of these mitochondria revealed that selectively permeabilized mitochondria were separate from the mitochondria network, suggesting that minority MOMP preferentially occurs on fragmented mitochondria (Figures 3B and 3C). Extensive mitochondrial fission is a well-established consequence of MOMP (Bhola et al., 2009; Frank et al., 2001). Therefore, to approach whether mitochondrial fragmentation was a cause or consequence of minority MOMP, U2OS cells expressing cytoGFP and mito-mCherry were imaged by live-cell microscopy. Treatment with ABT-737 (10 μM) led to minority MOMP, apparent by the translocation of cytoGFP into mitochondria after 124 min. Importantly, these mitochondria were fragmented from the mitochondrial network prior to cytoGFP translocation at 120 min (Figure 3D; Video S1). This suggests that minority MOMP preferentially occurs on fragmented mitochondria. We next used these cells to investigate a role for mitochondrial fusion in regulating minority MOMP. *Mfn1/2*$^{-/-}$ and *Mfn1/2*$^{-/-}$ + MFN2 MEF expressing the MOMP reporter were treated with a sub-lethal dose of ABT-737. Strikingly, increased levels of minority MOMP were observed in *Mfn1/2*$^{-/-}$ MEF when compared with *Mfn1/2*$^{-/-}$ + MFN2 MEF (Figure 3E; Figure S4A). This is consistent with minority MOMP occurring primarily on fragmented mitochondria, with mitochondrial fusion having an inhibitory effect. To further address this, we investigated the impact of inhibiting mitochondrial fission upon minority MOMP following treatment of *Drp1*$^{fl/fl}$ and *Drp1*$^{-/-}$ MEF with ABT-737. MEF expressing DRP1 undergo minority MOMP after exposure to ABT-737, but this was completely abolished in DRP1-deleted cells (Figure 3F; Figure S4B). Together, these data demonstrate that mitochondrial dynamics regulate minority MOMP; mitochondrial fusion appears to be inhibitory whereas fission promotes minority MOMP.

Pro-survival BCL-2 proteins display inter-mitochondrial heterogeneity in localization

Our data demonstrate that mitochondrial fission promotes minority MOMP, enabling caspase-dependent DNA damage. Nevertheless, how mitochondrial dynamics regulate minority MOMP is not known. Mitochondrial outer membrane integrity is regulated by the balance of pro- and anti-apoptotic BCL-2 family proteins (Campbell and Tait, 2018). We hypothesized that inter-mitochondrial variation in BCL-2 family localization may underlie minority MOMP. To investigate this hypothesis, we set out to visualize endogenous levels of BCL-2 family proteins on individual mitochondria. CRISPR-Cas9 genome editing can be used to knock in fluorescent proteins at defined genomic loci to enable endogenous tagging of proteins (Bukhari and Müller, 2019). Using this approach, we generated clonal knockin HeLa cell lines, where the red fluorescent protein Scarlet was fused to the N termini of BCL-2, BCL-xL, and MCL-1. As verification, western blotting using antibodies specific to BCL-2, BCL-xL, MCL-1, and Scarlet confirmed that these cell lines expressed these fusion proteins at similar levels to their endogenous counterparts (Figures 4A and 4B). Secondly, Airyscan super-resolution microscopy demonstrated mitochondrial localization of Scarlet-BCL-2, BCL-xL, and MCL-1, as expected (Figure 4C), and is comparable to mitochondrial localization of untagged endogenous BCL-2 proteins in parental cells (Figures S5A and S5B). Finally, we monitored cell viability using SYTOX Green exclusion and IncuCyte real-time imaging in response to BH3-mimetic treatment (ABT-737 and S63845). This demonstrated that all knockin cell lines underwent cell death in response to BH3-mimetic treatment (Figure S5C). Using these knockin cells, we next acquired super-resolution microscopy images of Scarlet-tagged BCL-2, BCL-xL, and MCL-1, and then applied a color-grading lookup table (LUT) such that the brighter the Scarlet signal, the brighter the image. This revealed heterogeneity of Scarlet-BCL-2, BCL-xL, and MCL-1 across the

(C) Quantification of fragmentation or elongation of mitochondria which have undergone minority MOMP, as visualized in (B). Data represented as mean (SD) from 4 independent experiments and analyzed using Student's t test.

(D) Live-cell Airyscan imaging of U2OS cells transfected with cytoGFP (green) and mito-mCherry (red), and treated with 10 μM ABT-737 in the presence of dimerizer. Scale bars, 10 μm.

See Video S1.

(E) Quantification of minority MOMP assessed in *Mfn1/2*$^{-/-}$ and *Mfn1*$^{-/-}$ MEF, transfected with cyto-GFP and mito-mCherry. Cells were treated with 10 μM ABT-737 for 3 h in the presence of dimerizer. Data represented as mean (SD) from 3 independent experiments.

(F) Quantification of minority MOMP assessed in WT and *Drp1*$^{fl/fl}$ MEF, transfected with cyto-GFP and mito-mCherry. Cells were treated with 10 μM ABT-737 for 3 h in the presence of dimerizer. Data represented as mean (SD) from 4 independent experiments.

Statistics: $^{*}p \leq 0.05$, $^{**}p \leq 0.01$, $^{***}p \leq 0.001$.

See also Figure S4.

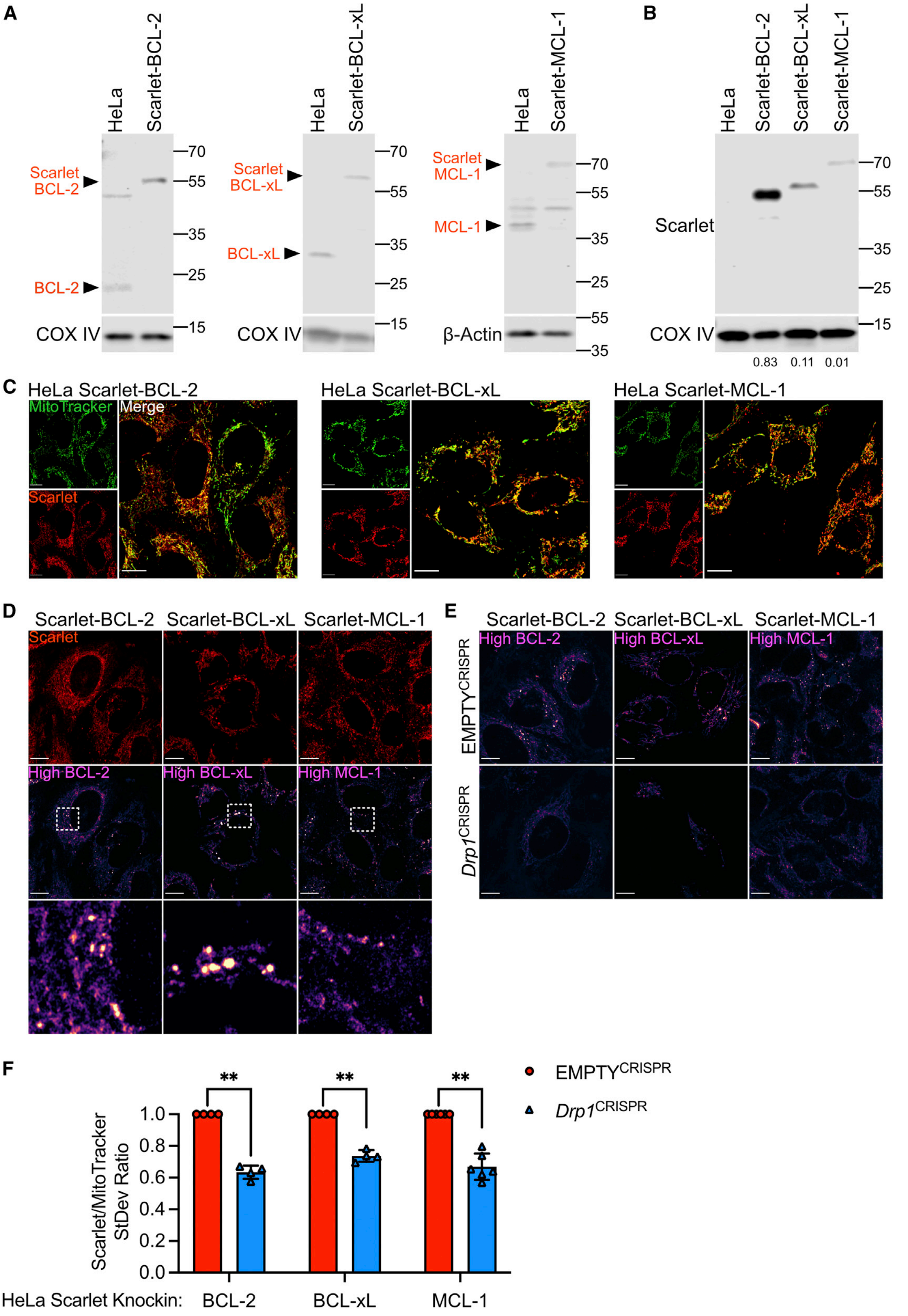

Figure 4. Pro-survival BCL-2 proteins display inter-mitochondrial heterogeneity in expression

(A) Immunoblots of HeLa cells with CRISPR-Cas9-mediated knockin of Scarlet into the BCL-2, BCL-xL, or MCL-1 locus using antibodies against BCL-2, BCL-xL, or MCL-1. COX IV or β-actin serves as loading controls.

(legend continued on next page)

mitochondrial network, which is also evident in parental cells stained with BCL-2 family antibodies (Figure 4D; Figure S5A). Given our previous data, we hypothesized that BCL-2 family protein heterogeneity is regulated by mitochondrial dynamics. To test this, we inhibited mitochondrial fission through CRISPR-Cas9 deletion of DRP1. Western blot confirmed DRP1 deletion, resulting in extensive mitochondrial hyperfusion (Figures S5D and S5E). Strikingly, cells with hyperfused mitochondria displayed much reduced inter-mitochondrial heterogeneity of MCL-1, BCL-2, or BCL-xL (Figures 4E and 4F; Figure S5F). Combined, these data show that within a cell, extensive inter-mitochondrial heterogeneity in BCL-2 localization exists that is impacted by mitochondrial dynamics.

Heterogeneity in apoptotic priming underpins minority MOMP

We next investigated whether there was a relationship between localization of anti-apoptotic BCL-2 proteins and minority MOMP. To determine this, we acquired super-resolution images of HeLa cells expressing endogenous Scarlet-BCL-2, BCL-xL, or MCL-1, together with OMI-GFP and MitoTracker Deep Red. During MOMP, soluble intermembrane space proteins, including Omi, are released from mitochondria (Bock and Tait, 2020). Mitochondria retain MitoTracker Deep Red even after loss of mitochondrial integrity; thus, mitochondria that have undergone MOMP are identifiable by loss of Omi and MitoTracker retention. Surprisingly, live-cell imaging of BCL-2 family protein knockin cells following treatment with ABT-737 revealed that mitochondria (determined by MitoTracker positivity) that release OMI-GFP have higher levels of BCL-2, BCL-xL, or MCL-1 local mitochondrial levels (Figures 5A–5C). Computational segmentation allowed us to distinguish BCL-2 family protein mitochondrial localization on MitoTracker positive structures which lack OMI expression, confirming that these mitochondria have indeed undergone minority MOMP. Quantification across a number of cells shows that mitochondria which undergo minority MOMP have increased BCL-2 family protein mitochondrial localization (Figures 5D–5F). Furthermore, line scans revealed regions of the mitochondrial network with high BCL-2 family residency but low OMI expression, that is, mitochondria which have likely undergone minority MOMP (Figures S6A and S6B). Unexpectedly, these data reveal a correlation between increased anti-apoptotic BCL-2 mitochondrial localization and selective mitochondrial permeabilization. We reasoned that this may be analogous to increased apoptotic priming at the cellular level, where high anti-apoptotic BCL-2 mitochondrial localization can correlate with apoptotic sensitivity in some cell types. Mitochondrial association of pro-apoptotic BAX is indicative of increasing apoptotic priming (Edlich et al., 2011; Reichenbach et al., 2017; Schellenberg et al., 2013). To investigate whether mitochondria with high BCL-2 local mitochondrial localization may also display high BAX localization (indicative of selective, increased apoptotic priming), we generated GFP-BAX expressing BCL-2 family knockin HeLa cells and imaged them by super-resolution microscopy. In line with the notion that mitochondria with higher BCL-2 family localization also have elevated BAX localization, we observed BAX co-localizing with high BCL-2 mitochondria, indicative of increased apoptotic priming (Figures 5G–5I). These data demonstrate that inter-mitochondrial heterogeneity in anti-apoptotic BCL-2 mitochondrial localization and apoptotic priming underlies minority MOMP.

Mitochondrial dysfunction promotes BAX accumulation promoting minority MOMP

We aimed to define the underlying mechanism of mitochondrial-intrinsic apoptotic priming. In healthy cells, BAX undergoes mitochondrial retrotranslocation, and inhibiting this process causes BAX mitochondrial accumulation, sensitizing to MOMP (Edlich et al., 2011; Schellenberg et al., 2013). HeLa cells expressing GFP-BAX and iRFP-Omp25 were treated with the uncoupler CCCP to induce mitochondrial dysfunction. To facilitate visualization of mitochondrial localized GFP-BAX, cells were treated with digitonin to selectively permeabilize the plasma membrane, as described previously (Bender et al., 2012). Inducing mitochondrial dysfunction by CCCP treatment led to robust mitochondrial recruitment of GFP-BAX (Figure 6A; Figure S7A; Videos S2, S3, S4, and S5). Immunostaining of HeLa cells with the activation-specific BAX antibody 6A7 revealed BAX activation, as expected, under conditions of apoptosis (combined BH3-mimetic treatment) but not following CCCP treatment (Figure S7B). Since mitochondrial fusion promotes efficient oxidative phosphorylation, reducing heterogeneity in mitochondrial function (Chen et al., 2003), we hypothesized that by impacting mitochondrial function, mitochondrial fission may promote BAX recruitment, thereby facilitating minority MOMP. We imaged *Mfn1/2*$^{-/-}$ and *Mfn1/2*$^{-/-}$ + MFN2 MEF with MitoTracker Red, a potentiometric dye, which reports mitochondrial $\Delta\psi^{m}$ as a measure of mitochondrial function. Consistent with defective mitochondrial function, mitochondria in fusion-defective cells (*Mfn1/2*$^{-/-}$) displayed a heterogenous MitoTracker Red signal and lower total signal than fusion competent *Mfn1/2*$^{-/-}$ + MFN2 MEF (Figures 6B–6D). We next analyzed GFP-BAX localization in *Mfn1/2*$^{-/-}$ and *Mfn1/2*$^{-/-}$ + MFN2 MEF, using fluorescence loss in photobleaching (FLIP) to help visualize mitochondrial localized GFP-BAX. FLIP

(B) Immunoblots of HeLa cells with CRISPR-Cas9-mediated knockin of Scarlet into the BCL-2, BCL-xL, or MCL-1 locus, using an antibody against Scarlet. COX IV serves as a loading control. Band quantification relative to COX IV loading shown below.

(C) Live-cell Airyscan imaging of HeLa Scarlet-BCL-2, Scarlet-BCL-xL, and Scarlet-MCL-1 (red) cells. Cells were incubated with MitoTracker Green (green) to stain mitochondria. Scale bars, 10 μm.

(D) Live-cell Airyscan imaging of HeLa Scarlet-BCL-2, Scarlet-BCL-xL, and Scarlet-MCL-1 (red) cells. Magenta LUT applied to reveal areas of high BCL-2, BCL-XL, and MCL-1 expression. Scale bars, 10 μm.

(E) Live-cell Airyscan imaging of HeLa Scarlet-BCL-2, Scarlet-BCL-xL, and Scarlet-MCL-1 cells, with and without CRISPR-Cas9-mediated *Drp1* deletion. Magenta LUT applied to reveal areas of high BCL-2, BCL-XL, and MCL-1 expression. Scale bars, 10 μm.

(F) Quantification of Scarlet to MitoTracker signal standard deviation in HeLa Scarlet-BCL-2, Scarlet-BCL-xL, and Scarlet-MCL-1 cells, with and without CRISPR-Cas9-mediated *Drp1* deletion. Data are expressed as mean (SD) from 4 to 5 independent experiments and analyzed using Student's t test.

Statistics: *p $\leq$ 0.05, **p $\leq$ 0.01, ***p $\leq$ 0.001.

See also Figure S5.

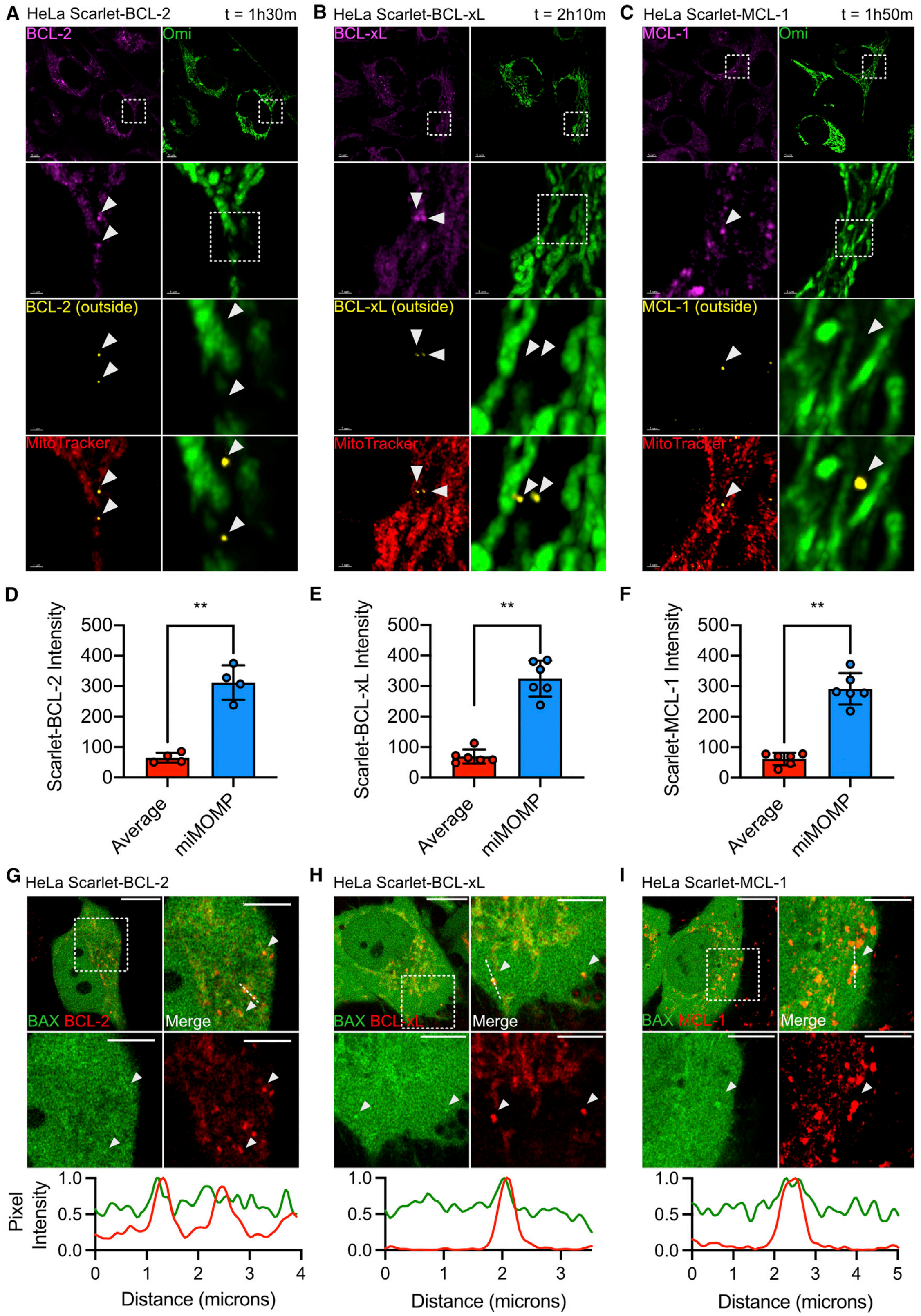

(legend on next page)

analysis of GFP-BAX revealed slower mitochondrial retrotranslocation in *Mfn1/2*$^{-/-}$ cells compared with *Mfn1/2*$^{-/-}$ + MFN2 cells (Figures 6E–6H; Videos S6 and S7). This suggests that mitochondrial dysfunction, a consequence of defective mitochondrial dynamics, can promote GFP-BAX mitochondrial accumulation, serving as a mitochondrial-intrinsic priming mechanism that facilitates minority MOMP.

DISCUSSION

We describe that mitochondrial dysfunction, inducing mitochondrial fission, promotes DNA damage and genome instability. This process requires caspase activity, which is engaged by minority MOMP, to trigger DNA damage. Investigating the underlying mechanism, we find that mitochondrial dynamics affect the inter-mitochondrial heterogeneity of anti-apoptotic BCL-2, permitting increased apoptotic priming of fragmented mitochondria. Mitochondrial dysfunction acts as a mitochondrial-intrinsic priming signal by inhibiting pro-apoptotic BAX retrotranslocation, promoting minority MOMP. Unexpectedly, by affecting mitochondrial BCL-2 heterogeneity and apoptotic priming, our data reveal crucial roles for mitochondrial dysfunction and dynamics in the regulation of minority MOMP leading to caspase-dependent DNA damage and genome instability.

Our study highlights that mitochondrial dynamics are integral to minority MOMP, whereby mitochondrial fusion inhibits and fission promotes this process. Consistent with this finding, the ability of sub-lethal apoptotic stress to engage oncogenic caspase-dependent DNA damage and genome instability was regulated in a similar manner. Moreover, we found in some cancer types, a correlation between the expression of the mitochondrial fission protein DRP1, DNA damage, and mutational burden. These data support an oncogenic role for mitochondrial fission through its capacity to promote minority MOMP and associated sub-lethal caspase activity. This also suggests that the multitude of cellular signaling pathways and stresses that impact mitochondrial dynamics—for instance, as hypoxia or high glycolytic rates—might facilitate minority MOMP-induced transformation (Chen and Chan, 2017; Wu et al., 2016). Indeed, we found that enforced mitochondrial fission (through MFN1/2 deletion) promoted minority MOMP-induced transformation. In line with previous findings, we also report that DRP1 supports oncogene-induced transformation—, minority MOMP may play a contributory role (Serasinghe et al., 2015). Our study underscores that extensive interplay exists between mitochondrial dynamics and cancer (Chen and Chan, 2017; Gao et al., 2017; Kashatus et al., 2015; Serasinghe et al., 2015; Zhao et al., 2013).

We sought to define how mitochondrial dynamics might control minority MOMP. Surprisingly, we found inter-mitochondrial heterogeneity in anti-apoptotic BCL-2 localization. This heterogeneity was suppressed by mitochondrial fusion, most likely because mitochondrial fusion enables homogeneous distribution of BCL-2 proteins across the mitochondrial network. As we further discuss, heterogeneity in anti-apoptotic BCL-2 localization enables differences in apoptotic priming of specific mitochondria. Interestingly, during cell death, mitochondrial variation in pro-apoptotic BAK levels have previously been found to influence the kinetics of MOMP (Weaver et al., 2014). Though myriad interconnections between mitochondrial dynamics and apoptosis exist, mitochondrial fission is largely considered a consequence of cell death. For instance, during apoptosis, extensive mitochondrial fragmentation occurs subsequent to MOMP (Bhola et al., 2009). By promoting homogeneous BCL-2 localization across the mitochondrial network, our data reveal an indirect role for mitochondrial fusion in preventing minority MOMP.

We have previously found that ectopic expression of BCL-2 can lead to incomplete MOMP, consistent with BCL-2 anti-apoptotic function (Tait et al., 2010). In the current study, we find that increased local mitochondrial levels of anti-apoptotic BCL-2 family proteins correlates with selective mitochondrial permeabilization. Although this may seem initially counter-intuitive, precedence for increased apoptotic priming, correlating with high anti-apoptotic BCL-2 levels, is evident in various cancers (Certo et al., 2006; Singh et al., 2019). This is perhaps best demonstrated in high-BCL-2-expressing chronic lymphocytic leukemia (CLL) that is often highly sensitive to the BCL-2-selective BH3-mimetic, venetoclax (Roberts et al., 2016). In healthy cells, BAX mitochondrial localization is indicative of increased apoptotic priming (Edlich et al., 2011; Kuwana et al., 2020; Reichenbach et al., 2017; Schellenberg et al., 2013).

Figure 5. Heterogeneity in apoptotic priming underpins minority MOMP

(A) Live-cell Airyscan imaging of HeLa Scarlet-BCL-2 (magenta) transfected with Omi-GFP (green) and incubated with MitoTracker Deep Red (red). Cells were treated with 10 μM ABT-737 for the time indicated. Images were processed with Imaris to determine BCL-2, BCL-xL, or MCL-1 expression (yellow) at mitochondrial areas lacking Omi-GFP expression. Scale bar as indicated on images.

(B) HeLa Scarlet-BCL-xL imaged as (A).

(C) HeLa Scarlet-MCL-1 imaged as (A).

(D) Quantification of Scarlet-BCL-2 intensity at mitochondrial regions determined by MitoTracker Deep Red staining but lacking Omi-GFP. Data are expressed as mean (SD) and analyzed using Student's t test.

(E) HeLa Scarlet-BCL-xL quantified as (D).

(F) HeLa Scarlet-MCL-1 quantified as (D).

(G) Live-cell Airyscan imaging of HeLa Scarlet-BCL-2 (red) cells stably overexpressing GFP-BAX (green). Arrows indicate regions of high BCL-2 expression with high GFP-BAX expression. Scale bars, 10 μm. Dotted line represents pixel intensities for the dotted track shown, GFP-BAX in green and BCL-2 in red.

(H) Live-cell Airyscan images of HeLa Scarlet-BCL-xL (red) cells stably overexpressing GFP-BAX (green). Arrows indicate regions of high BCL-xL expression with high GFP-BAX expression. Scale bars, 10 μm. Dotted line represents pixel intensities for the dotted track shown, GFP-BAX in green and BCL-xL in red.

(I) Live-cell Airyscan images of HeLa Scarlet-MCL-1 (red) cells stably overexpressing GFP-BAX (green). Arrows indicate regions of high MCL-1 expression with high GFP-BAX expression. Scale bars, 10 μm. Dotted line represents pixel intensities for the dotted track shown, GFP-BAX in green and MCL-1 in red.

Statistics: *$p \leq 0.05$, **$p \leq 0.01$, ***$p \leq 0.001$.

See also Figure S6.

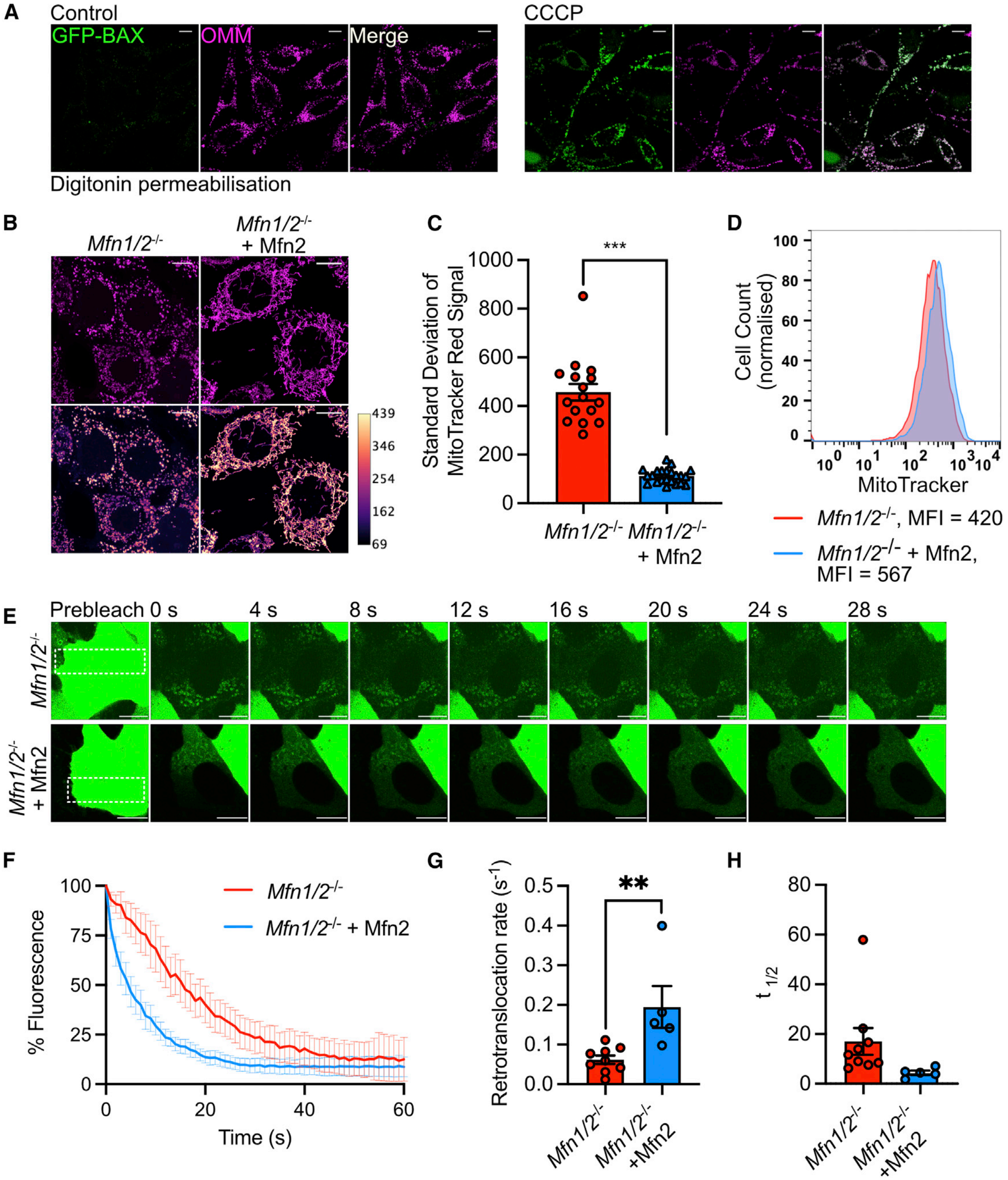

Figure 6. Mitochondrial dysfunction promotes BAX accumulation on mitochondria promoting minority MOMP

(A) HeLa cells stably expressing GFP-BAX (green) and Omp25-iRFP (outermitochondrial membrane {OMM}, magenta), treated with and without 10 μM of CCCP for 30 min prior to digitonin permeabilization.

See Videos S2 and S3.

(B) *Mfn1/2*$^{-/-}$ and *Mfn1/2*$^{-/-}$ + MFN2 MEF pulsed with MitoTracker Red and imaged. Images with magenta LUT applied are shown in lower panels. Scale bars, 10 μm. Data are representative from three independent experiments.

(C) Standard deviation of MitoTracker Red signal in *Mfn1/2*$^{-/-}$ and *Mfn1/2*$^{-/-}$ + MFN2 MEF pulsed with MitoTracker Red acquired using Airyscan. Data are expressed as mean ± SEM from three independent experiments and analyzed using Student's t test.

(D) Fluorescence profiles of *Mfn1/2*$^{-/-}$ and *Mfn1/2*$^{-/-}$ + MFN2 MEF pulsed with MitoTracker Red. Data are representative of 2 independent experiments.

(E) *Mfn1/2*$^{-/-}$ and *Mfn1/2*$^{-/-}$ + Mfn2 MEF stably expressing GFP-BAX (green) imaged pre- and post-bleaching to reveal mitochondrially localized GFP-BAX. Scale bars, 10 μm.

See Videos S6 and S7.

(F) FLIP analysis of *Mfn1/2*$^{-/-}$ and *Mfn1/2*$^{-/-}$ + MFN2 MEF stably overexpressing GFP-BAX. Mitochondrial GFP-BAX dissociation was imaged over 60 s and the first image post-bleaching is set to 0 s. Data are expressed as mean ± SEM. *Mfn1/2*$^{-/-}$ n = 9 cells; *Mfn1/2*$^{-/-}$ + MFN2 n = 5 cells.

(legend continued on next page)

Indeed, further investigation revealed that high pro-apoptotic BAX localization correlated with high BCL-2 localization on mitochondria. Our data argue that heterogeneity in apoptotic priming exists not only between cell types but also intracellularly at the level of individual mitochondria.

Finally, we sought to understand how inter-mitochondrial heterogeneity in apoptotic priming might occur. Pro-apoptotic BAX is subject to constant mitochondrial retrotranslocation; inhibition of BAX retrotranslocation leads to mitochondrial accumulation, sensitizing to apoptosis (Edlich et al., 2011; Schellenberg et al., 2013). We find that reduction of mitochondrial inner membrane potential ($\Delta\psi_m$) promotes BAX mitochondrial localization. Importantly, reduction of $\Delta\psi_m$, provides a mitochondrial-intrinsic signal to increase apoptotic priming. Under conditions of mitochondrial dysfunction imposed by loss of mitochondrial fusion, we find decreased rates of BAX retrotranslocation, enabling its mitochondrial accumulation. In essence, BAX retrotranslocation may serve as a barometer of cellular metabolic health. Because loss of mitochondrial function causes mitochondrial fission, it promotes minority MOMP in a 2-fold manner, segregating dysfunctional mitochondria and promoting BAX accumulation (Twig et al., 2008). Further investigation will be required to mechanistically delineate how mitochondrial function regulates BAX retrotranslocation. We consider it likely that additional mechanisms of mitochondrial-intrinsic priming also exist; for instance, levels of anti-apoptotic BCL-2 mitochondrial localization will dictate a given cell's propensity to engage minority MOMP. Moreover, degradation of dysfunctional mitochondria through mitophagy, may also impact the occurrence of minority MOMP. Indeed, others have reported that permeabilized mitochondria are targeted for mitophagy—this likely affects the steady-state detection of minority MOMP (Lindqvist et al., 2018).

Our study investigates the impact of mitochondrial dynamics and dysfunction upon BH3-mimetic-induced minority MOMP. When might this occur physiologically? One possibility is during intracellular bacterial infection (*Salmonella* Typhimurium and *Chlamydia trachomatis*), where minority MOMP has been shown to cause inflammation as an innate immune response (Brokatzky et al., 2019). Interestingly, although *C. trachomatis* actively promotes mitochondrial fusion during early infection, late-stage infection is associated with mitochondrial fragmentation, providing an ideal setting to engage minority MOMP-induced inflammation (Kurihara et al., 2019; Liang et al., 2018). More recently, mtDNA damage has been shown to promote minority MOMP, enabling mitochondrial-nuclear retrograde signaling (Tigano et al., 2021). In this setting, mitochondrial fragmentation most likely enables some mitochondria to selectively permeabilize in response to mtDNA damage. A final example may relate to minority MOMP that we have previously observed under homeostatic conditions (Ichim et al., 2015). Indeed, others have found basal levels of caspase/CAD-dependent DNA damage in cycling cells (Liu et al., 2017). Given that extensive mitochondrial fission occurs during mitosis, this may serve to underpin basal levels of minority MOMP and caspase-dependent DNA damage.

In summary, our findings that reveal that mitochondrial dynamics regulate DNA damage and genome instability via minority MOMP-induced caspase activity. This provides a mechanism linking mitochondrial dysfunction to pro-oncogenic DNA damage. Beyond pro-tumorigenic effects, minority MOMP has also been shown to have roles in innate immunity and inflammation; as such, our findings suggest new approaches to modulate minority MOMP and its downstream functions.

Limitations of the study

In this study, we investigated the impact of mitochondrial function and dynamics on DNA damage and genome instability. This was done under chemically induced mitochondrial dysfunction or through genetic perturbation of mitochondrial dynamics. Future work should investigate whether pathophysiological loss of mitochondrial function promotes DNA damage through sublethal apoptotic stress. Additionally, our experiments were carried out *in vitro* using cell culture, thus it will be important to validate these findings in an *in vivo* setting.

STAR★METHODS

Detailed methods are provided in the online version of this paper and include the following:

- KEY RESOURCES TABLE
- RESOURCE AVAILABILITY
  - Lead contact
  - Materials availability
  - Data and code availability
- EXPERIMENTAL MODEL AND SUBJECT DETAILS
- METHOD DETAILS
  - Generation of Scarlet-BCL-2 knock-in cell lines
  - Generation of stable overexpressing cell lines
  - Generation of CRISPR knock-out cell lines
  - Plasmids
  - Western blotting
  - Flow cytometry
  - PALA assay and Cad genomic amplification
  - Anchorage-independent growth assay
  - qPCR
  - Microscopy
  - Live-cell viability assays
  - Bioinformatic analysis

SUPPLEMENTAL INFORMATION

Supplemental information can be found online at https://doi.org/10.1016/j.devcel.2022.03.019.

ACKNOWLEDGMENTS

This research was supported by funding from the Cancer Research UK (A20145; S.W.G.T.). The Wellcome Centre for Cell-Matrix Research is supported by Wellcome Trust (088785/Z/09/Z). R.H. is supported by a fellowship

(G) Retrotranslocation rates from cells analyzed in (F). Data expressed as mean ± SEM and analyzed using Student's t test.
(H) Half-life ($t_{1/2}$) of GFP-BAX from cells analyzed in (F). Data expressed as mean ± SEM and analyzed using Student's t test.
Statistics: *p ≤ 0.05, **p ≤ 0.01, ***p ≤ 0.001.
See also Figure S7.

from the Swiss National Science Foundation. We thank Douglas Green (St. Jude Children's Research Hospital), David Chan (Caltech), and Hiromi Sesaki (Johns Hopkins University) for reagents. We also thank Margaret O'Prey, Nikki Paul, Peter Thomason, and Tom Gilbey (Beatson Institute) for excellent technical assistance, Catherine Winchester (Beatson Institute), Douglas Green, and members of the Tait laboratory for critical reading of the manuscript.

AUTHOR CONTRIBUTIONS

Conceived the study and designed the work plan, K.C., J.S.R., and S.W.G.T.; experimental work, K.C., J.S.R., R.H., A.E.M.-G., E.V., K.B., C.C., Y.E., D.G.S., G.I., K.J.C., and A.P.G.; development and contribution of reagents, K.C., J.S.R., C.C., and G.I.; data analysis, K.C., J.S.R., Y.E., K.J.C., A.P.G., and S.W.G.T.; intellectual input, K.C., J.S.R., K.J.C., A.P.G., and S.W.G.T.; manuscript writing, J.S.R. and S.W.G.T.

DECLARATION OF INTERESTS

The authors declare no competing interests.

INCLUSION AND DIVERSITY

One or more of the authors of this paper self-identifies as a member of the LGBTQ+ community.

Received: May 12, 2021
Revised: February 22, 2022
Accepted: March 30, 2022
Published: April 20, 2022

REFERENCES

Bender, C.E., Fitzgerald, P., Tait, S.W., Llambi, F., McStay, G.P., Tupper, D.O., Pellettieri, J., Sánchez Alvarado, A., Salvesen, G.S., and Green, D.R. (2012). Mitochondrial pathway of apoptosis is ancestral in metazoans. Proc. Natl. Acad. Sci. USA *109*, 4904–4909.

Berthenet, K., Castillo Ferrer, C., Fanfone, D., Popgeorgiev, N., Neves, D., Bertolino, P., Gibert, B., Hernandez-Vargas, H., and Ichim, G. (2020). Failed apoptosis enhances melanoma cancer cell aggressiveness. Cell Rep. *31*, 107731.

Bhola, P.D., Mattheyses, A.L., and Simon, S.M. (2009). Spatial and temporal dynamics of mitochondrial membrane permeability waves during apoptosis. Biophys. J. *97*, 2222–2231.

Bock, F.J., and Tait, S.W.G. (2020). Mitochondria as multifaceted regulators of cell death. Nat. Rev. Mol. Cell Biol. *21*, 85–100.

Brokatzky, D., Dörflinger, B., Haimovici, A., Weber, A., Kirschnek, S., Vier, J., Metz, A., Henschel, J., Steinfeldt, T., Gentle, I.E., and Häcker, G. (2019). A non-death function of the mitochondrial apoptosis apparatus in immunity. EMBO J. *38*, e100907.

Bukhari, H., and Müller, T. (2019). Endogenous fluorescence tagging by CRISPR. Trends Cell Biol. *29*, 912–928.

Campbell, K.J., and Tait, S.W.G. (2018). Targeting BCL-2 regulated apoptosis in cancer. Open Biol. *8*, 180002.

Cerami, E., Gao, J., Dogrusoz, U., Gross, B.E., Sumer, S.O., Aksoy, B.A., Jacobsen, A., Byrne, C.J., Heuer, M.L., Larsson, E., et al. (2012). The cBio cancer genomics portal: an open platform for exploring multidimensional cancer genomics data. Cancer Discov. *2*, 401–404.

Certo, M., Del Gaizo Moore, V., Nishino, M., Wei, G., Korsmeyer, S., Armstrong, S.A., and Letai, A. (2006). Mitochondria primed by death signals determine cellular addiction to antiapoptotic BCL-2 family members. Cancer Cell *9*, 351–365.

Chen, E.Y., Tan, C.M., Kou, Y., Duan, Q., Wang, Z., Meirelles, G.V., Clark, N.R., and Ma'ayan, A. (2013). Enrichr: interactive and collaborative HTML5 gene list enrichment analysis tool. BMC Bioinformatics *14*, 128.

Chen, H., and Chan, D.C. (2017). Mitochondrial dynamics in regulating the unique phenotypes of cancer and stem cells. Cell Metab. *26*, 39–48.

Chen, H., Detmer, S.A., Ewald, A.J., Griffin, E.E., Fraser, S.E., and Chan, D.C. (2003). Mitofusins Mfn1 and Mfn2 coordinately regulate mitochondrial fusion and are essential for embryonic development. J. Cell Biol. *160*, 189–200.

de Brito, O.M., and Scorrano, L. (2008). Mitofusin 2 tethers endoplasmic reticulum to mitochondria. Nature *456*, 605–610.

Edlich, F., Banerjee, S., Suzuki, M., Cleland, M.M., Arnoult, D., Wang, C., Neutzner, A., Tjandra, N., and Youle, R.J. (2011). Bcl-x(L) retrotranslocates Bax from the mitochondria into the cytosol. Cell *145*, 104–116.

Frank, S., Gaume, B., Bergmann-Leitner, E.S., Leitner, W.W., Robert, E.G., Catez, F., Smith, C.L., and Youle, R.J. (2001). The role of dynamin-related protein 1, a mediator of mitochondrial fission, in apoptosis. Dev. Cell *1*, 515–525.

Gao, J., Aksoy, B.A., Dogrusoz, U., Dresdner, G., Gross, B., Sumer, S.O., Sun, Y., Jacobsen, A., Sinha, R., Larsson, E., et al. (2013). Integrative analysis of complex cancer genomics and clinical profiles using the cBioPortal. Sci. Signal. *6*, pl1.

Gao, Z., Li, Y., Wang, F., Huang, T., Fan, K., Zhang, Y., Zhong, J., Cao, Q., Chao, T., Jia, J., et al. (2017). Mitochondrial dynamics controls anti-tumor innate immunity by regulating CHIP-IRF1 axis stability. Nat. Commun. *8*, 1805.

Giampazolias, E., and Tait, S.W. (2016). Mitochondria and the hallmarks of cancer. FEBS J. *283*, 803–814.

Goldstein, J.C., Waterhouse, N.J., Juin, P., Evan, G.I., and Green, D.R. (2000). The coordinate release of cytochrome *c* during apoptosis is rapid, complete and kinetically invariant. Nat. Cell Biol. *2*, 156–162.

Gorelick, A.N., Kim, M., Chatila, W.K., La, K., Hakimi, A.A., Berger, M.F., Taylor, B.S., Gammage, P.A., and Reznik, E. (2021). Respiratory complex and tissue lineage drive recurrent mutations in tumor mtDNA. Nat. Metab. *3*, 558–570.

Ichim, G., Lopez, J., Ahmed, S.U., Muthalagu, N., Giampazolias, E., Delgado, M.E., Haller, M., Riley, J.S., Mason, S.M., Athineos, D., et al. (2015). Limited mitochondrial permeabilization causes DNA damage and genomic instability in the absence of cell death. Mol. Cell *57*, 860–872.

Ichim, G., and Tait, S.W. (2016). A fate worse than death: apoptosis as an oncogenic process. Nat. Rev. Cancer *16*, 539–548.

Isaacs, J.S., Jung, Y.J., Mole, D.R., Lee, S., Torres-Cabala, C., Chung, Y.L., Merino, M., Trepel, J., Zbar, B., Toro, J., et al. (2005). HIF overexpression correlates with biallelic loss of fumarate hydratase in renal cancer: novel role of fumarate in regulation of HIF stability. Cancer Cell *8*, 143–153.

Ishihara, N., Nomura, M., Jofuku, A., Kato, H., Suzuki, S.O., Masuda, K., Otera, H., Nakanishi, Y., Nonaka, I., Goto, Y., et al. (2009). Mitochondrial fission factor Drp1 is essential for embryonic development and synapse formation in mice. Nat. Cell Biol. *11*, 958–966.

Kashatus, J.A., Nascimento, A., Myers, L.J., Sher, A., Byrne, F.L., Hoehn, K.L., Counter, C.M., and Kashatus, D.F. (2015). Erk2 phosphorylation of Drp1 promotes mitochondrial fission and MAPK-driven tumor growth. Mol. Cell *57*, 537–551.

Kuleshov, M.V., Jones, M.R., Rouillard, A.D., Fernandez, N.F., Duan, Q., Wang, Z., Koplev, S., Jenkins, S.L., Jagodnik, K.M., Lachmann, A., et al. (2016). Enrichr: a comprehensive gene set enrichment analysis web server 2016 update. Nucleic Acids Res. *44*, W90–W97.

Kurihara, Y., Itoh, R., Shimizu, A., Walenna, N.F., Chou, B., Ishii, K., Soejima, T., Fujikane, A., and Hiromatsu, K. (2019). *Chlamydia trachomatis* targets mitochondrial dynamics to promote intracellular survival and proliferation. Cell. Microbiol. *21*, e12962.

Kuwana, T., King, L.E., Cosentino, K., Suess, J., Garcia-Saez, A.J., Gilmore, A.P., and Newmeyer, D.D. (2020). Mitochondrial residence of the apoptosis inducer BAX is more important than BAX oligomerization in promoting membrane permeabilization. J. Biol. Chem. *295*, 1623–1636.

Lartigue, L., Kushnareva, Y., Seong, Y., Lin, H., Faustin, B., and Newmeyer, D.D. (2009). Caspase-independent mitochondrial cell death results from loss of respiration, not cytotoxic protein release. Mol. Biol. Cell *20*, 4871–4884.

Liang, P., Rosas-Lemus, M., Patel, D., Fang, X., Tuz, K., and Juárez, O. (2018). Dynamic energy dependency of *Chlamydia trachomatis* on host cell metabolism during intracellular growth: role of sodium-based energetics in chlamydial ATP generation. J. Biol. Chem. *293*, 510–522.

Lindqvist, L.M., Frank, D., McArthur, K., Dite, T.A., Lazarou, M., Oakhill, J.S., Kile, B.T., and Vaux, D.L. (2018). Autophagy induced during apoptosis degrades mitochondria and inhibits type I interferon secretion. Cell Death Differ. *25*, 784–796.

Liu, X., Li, F., Huang, Q., Zhang, Z., Zhou, L., Deng, Y., Zhou, M., Fleenor, D.E., Wang, H., Kastan, M.B., and Li, C.-Y. (2017). Self-inflicted DNA double-strand breaks sustain tumorigenicity and stemness of cancer cells. Cell Res. *27*, 764–783.

Lovric, M.M., and Hawkins, C.J. (2010). TRAIL treatment provokes mutations in surviving cells. Oncogene *29*, 5048–5060.

Oltersdorf, T., Elmore, S.W., Shoemaker, A.R., Armstrong, R.C., Augeri, D.J., Belli, B.A., Bruncko, M., Deckwerth, T.L., Dinges, J., Hajduk, P.J., et al. (2005). An inhibitor of Bcl-2 family proteins induces regression of solid tumours. Nature *435*, 677–681.

Pollard, P.J., Spencer-Dene, B., Shukla, D., Howarth, K., Nye, E., El-Bahrawy, M., Deheragoda, M., Joannou, M., McDonald, S., Martin, A., et al. (2007). Targeted inactivation of fh1 causes proliferative renal cyst development and activation of the hypoxia pathway. Cancer Cell *11*, 311–319.

Ran, F., Hsu, P., Wright, J., Argawal, V., Scott, D., and Zhang, F. (2013). Genome engineering using the CRISPR-Cas9 system. Nat. Protoc. *8*, 2281–2308. https://doi.org/10.1038/nprot.2013.143.

Rehm, M., Düssmann, H., and Prehn, J.H. (2003). Real-time single cell analysis of Smac/DIABLO release during apoptosis. J. Cell Biol. *162*, 1031–1043.

Reichenbach, F., Wiedenmann, C., Schalk, E., Becker, D., Funk, K., Scholz-Kreisel, P., Todt, F., Wolleschak, D., Döhner, K., Marquardt, J.U., et al. (2017). Mitochondrial BAX determines the predisposition to apoptosis in human AML. Clin. Cancer Res. *23*, 4805–4816.

Reya, T., Duncan, A., Ailles, L., Domen, J., Scherer, D., Willert, K., Hintz, L., Nusse, R., and Weissman, I. (2003). A role for Wnt signalling in self-renewal of haematopoietic stem cells. Nature *423*, 409–414. https://doi.org/10.1038/nature01593.

Roberts, A.W., Davids, M.S., Pagel, J.M., Kahl, B.S., Puvvada, S.D., Gerecitano, J.F., Kipps, T.J., Anderson, M.A., Brown, J.R., Gressick, L., et al. (2016). Targeting BCL2 with Venetoclax in relapsed chronic lymphocytic leukemia. N. Engl. J. Med. *374*, 311–322.

Sanjana, N., Shalem, O., and Zhang, F. (2014). Improved vectors and genome-wide libraries for CRISPR screening. Nat. Methods *11*, 783–784. https://doi.org/10.1038/nmeth.3047.

Schellenberg, B., Wang, P., Keeble, J.A., Rodriguez-Enriquez, R., Walker, S., Owens, T.W., Foster, F., Tanianis-Hughes, J., Brennan, K., Streuli, C.H., and Gilmore, A.P. (2013). Bax exists in a dynamic equilibrium between the cytosol and mitochondria to control apoptotic priming. Mol. Cell *49*, 959–971.

Schneider, C., Rasband, W., and Eliceiri, K. (2012). NIH Image to ImageJ: 25 years of image analysis. Nat. Methods *9*, 671–675. https://doi.org/10.1038/nmeth.2089.

Sciacovelli, M., Gonçalves, E., Johnson, T.I., Zecchini, V.R., da Costa, A.S., Gaude, E., Drubbel, A.V., Theobald, S.J., Abbo, S.R., Tran, M.G., et al. (2016). Fumarate is an epigenetic modifier that elicits epithelial-to-mesenchymal transition. Nature *537*, 544–547.

Selak, M.A., Armour, S.M., MacKenzie, E.D., Boulahbel, H., Watson, D.G., Mansfield, K.D., Pan, Y., Simon, M.C., Thompson, C.B., and Gottlieb, E. (2005). Succinate links TCA cycle dysfunction to oncogenesis by inhibiting HIF-alpha prolyl hydroxylase. Cancer Cell *7*, 77–85.

Serasinghe, M.N., Wieder, S.Y., Renault, T.T., Elkholi, R., Asciolla, J.J., Yao, J.L., Jabado, O., Hoehn, K., Kageyama, Y., Sesaki, H., and Chipuk, J.E. (2015). Mitochondrial division is requisite to RAS-induced transformation and targeted by oncogenic MAPK pathway inhibitors. Mol. Cell *57*, 521–536.

Singh, R., Letai, A., and Sarosiek, K. (2019). Regulation of apoptosis in health and disease: the balancing act of BCL-2 family proteins. Nat. Rev. Mol. Cell Biol. *20*, 175–193.

Smith, A.L., Whitehall, J.C., Bradshaw, C., Gay, D., Robertson, F., Blain, A.P., Hudson, G., Pyle, A., Houghton, D., Hunt, M., et al. (2020). Age-associated mitochondrial DNA mutations cause metabolic remodelling that contributes to accelerated intestinal tumorigenesis. Nat. Cancer *1*, 976–989.

Stewart, S., Dykxhoorn, D., Palliser, D., Mizuno, H., Yu, E., An, D., Sabatini, D., Chen, I., Hahn, W., Sharp, P., et al. (2003). Lentivirus-delivered stable gene silencing by RNAi in primary cells. RNA *9*, 493–501. https://doi.org/10.1261/rna.2192803.

Stewart-Ornstein, J., and Lahav, G. (2016). Dynamics of CDKN1A in single cells defined by an endogenous fluorescent tagging toolkit. Cell Rep. *14*, 1800–1811.

Tait, S.W., Parsons, M.J., Llambi, F., Bouchier-Hayes, L., Connell, S., Muñoz-Pinedo, C., and Green, D.R. (2010). Resistance to caspase-independent cell death requires persistence of intact mitochondria. Dev. Cell *18*, 802–813.

Tigano, M., Vargas, D.C., Tremblay-Belzile, S., Fu, Y., and Sfeir, A. (2021). Nuclear sensing of breaks in mitochondrial DNA enhances immune surveillance. Nature *591*, 477–481.

Twig, G., Elorza, A., Molina, A.J., Mohamed, H., Wikstrom, J.D., Walzer, G., Stiles, L., Haigh, S.E., Katz, S., Las, G., et al. (2008). Fission and selective fusion govern mitochondrial segregation and elimination by autophagy. EMBO J. *27*, 433–446.

Wahl, G.M., Padgett, R.A., and Stark, G.R. (1979). Gene amplification causes overproduction of the first three enzymes of UMP synthesis in N-(phosphonacetyl)-L-aspartate-resistant hamster cells. J. Biol. Chem. *254*, 8679–8689.

Wakabayashi, J., Zhang, Z., Wakabayashi, N., Tamura, Y., Fukaya, M., Kensler, T.W., Iijima, M., and Sesaki, H. (2009). The dynamin-related GTPase Drp1 is required for embryonic and brain development in mice. J. Cell Biol. *186*, 805–816.

Weaver, D., Eisner, V., Liu, X., Várnai, P., Hunyady, L., Gross, A., and Hajnóczky, G. (2014). Distribution and apoptotic function of outer membrane proteins depend on mitochondrial fusion. Mol. Cell *54*, 870–878.

Wu, W., Lin, C., Wu, K., Jiang, L., Wang, X., Li, W., Zhuang, H., Zhang, X., Chen, H., Li, S., et al. (2016). FUNDC1 regulates mitochondrial dynamics at the ER-mitochondrial contact site under hypoxic conditions. EMBO J. *35*, 1368–1384.

Zhao, J., Zhang, J., Yu, M., Xie, Y., Huang, Y., Wolff, D.W., Abel, P.W., and Tu, Y. (2013). Mitochondrial dynamics regulates migration and invasion of breast cancer cells. Oncogene *32*, 4814–4824.

STAR★METHODS

KEY RESOURCES TABLE

REAGENT or RESOURCE	SOURCE	IDENTIFIER
Antibodies		
Mouse monoclonal anti-pATM (Ser1981), clone 10H11.E12	Novus	Cat# NB100-307, RRID:AB_10002350
Rabbit polyclonal anti-Phospho-Histone H2A.X (Ser139), clone 20E3	Cell Signaling Technology	Cat# 9718, RRID:AB_2118009
Rabbit monoclonal anti-Mitofusin-2, clone D2D10	Cell Signaling Technology	Cat# 9482
Mouse monoclonal anti-α-Tubulin, clone B-5-1-2	Sigma-Aldrich	Cat# T5168, RRID:AB_477579
Rabbit polyclonal anti-β-tubulin	Cell Signaling Technology	Cat# 2146
Rabbit polyclonal TOM20	Proteintech	Cat# 11802-1-AP, RRID:AB_2207530
Rabbit polyclonal HSP60, clone D307	Cell Signaling Technology	Cat# 4870, RRID:AB_2295614
Rabbit monoclonal DRP1, clone D8H5	Cell Signaling Technology	Cat# 5391, RRID:AB_11178938
Rabbit monoclonal Mitofusin-1, clone EPR7960	Abcam	Cat# ab129154
GFP	N/A	N/A
Mouse monoclonal anti-β-actin, clone AC-74	Sigma-Aldrich	Cat# A5316, RRID:AB_476743
Rabbit monoclonal anti-Bcl-2, clone D55G8	Cell Signaling Technology	Cat# 4223, RRID:AB_1903909
Rabbit polyclonal anti-Bcl-xL	Cell Signaling Technology	Cat# 2762, RRID:AB_10694844
Rabbit monoclonal anti-Mcl-1, clone D35A5	Cell Signaling Technology	Cat# 5453, RRID:AB_10694494
Rabbit monoclonal anti-COX IV, clone 3E11	Cell Signaling Technology	Cat# 4850, RRID:AB_2085424
Mouse monoclonal anti-COX IV, clone 4D11-B3-E8	Cell Signaling Technology	Cat# 11967, RRID:AB_2797784
Mouse monoclonal anti-Bcl-2, clone C-2	Santa Cruz Biotechnology	Cat# sc-7382, RRID:AB_626736
Rabbit monoclonal anti-Bcl-xL (54H6)	Cell Signaling Technology	Cat# 2764, RRID:AB_2228008
Rabbit polyclonal anti-Mcl-1	Proteintech	Cat# 16225-1-AP, RRID:AB_2143977
Mouse monoclonal Alexa Fluor 647 anti-H2A.X phosphor (Ser139), clone 2F3	Biolegend	Cat# 613407, RRID:AB_2114994
Mouse monoclonal anti-RFP, clone 6G6	ChromoTek	Cat# 6g6-100, RRID:AB_2631395
Bacterial and virus strains		
Ad5CMVCre	Uni Iowa Viral Vector Core	Cat# VVC-U of Iowa-5
Chemicals, peptides, and recombinant proteins		
MitoTracker Deep Red	Thermo Fisher Scientific	Cat# M22426
MitoTracker Red CMXRos	Thermo Fisher Scientific	Cat# M7512
SYTOX Green Nucleic Acid Stain	Thermo Fisher Scientific	Cat# S7020

(*Continued on next page*)

Continued		
REAGENT or RESOURCE	SOURCE	IDENTIFIER
ABT-737	ApexBio	Cat# A8193
S63845	ApexBio	Cat# A8737
Carbonyl cyanide 3-chlorophenylhydrazone (CCCP)	Sigma-Aldrich	Cat# C2759
Q-VD-OPh hydrate (QVD)	Adooq Biosciences	Cat# A14915
L-Aspartic acid, N-(phosphonoacetyl)-, disodium salt (PALA)	NCI Developmental Therapeutics Program	Cat# 224131
Critical commercial assays		
Alt-R Genome Editing Detection Kit	IDT	Cat# 1075931
Experimental models: Cell lines		
Mouse embryonic fibroblast, MEF	Gift from D. Green	N/A
Mouse embryonic fibroblast, MEF *Mfn1/2*$^{-/-}$	Gift from D. Chan	N/A
Mouse embryonic fibroblast, MEF *Mfn1/2*$^{-/-}$ + Mfn2	This paper	N/A
Human endocervical adenocarcinoma, HeLa	ATCC	Cat# CRL-7923, RRID:CVCL_0030
Human osteosarcoma, U2OS	ATCC	Cat# 300364/ p489_U-2_OS, RRID:CVCL_0042
Mouse embryonic fibroblast, MEF *Drp1*$^{fl/fl}$	Gift from H. Sesaki	N/A
Mouse embryonic fibroblast, MEF LZRS	This paper	N/A
Mouse embryonic fibroblast, MEF LZRS-DRP1	This paper	N/A
Human embryonic kidney, HEK293-FT	Thermo Fisher	RRID:CVCL_6911
HeLa Scarlet-BCL-2 knockin	This paper	N/A
HeLa Scarlet-BCL-xL knockin	This paper	N/A
HeLa Scarlet-MCL-1 knockin	This paper	N/A
Mouse embryonic fibroblast, MEF loxP-STOP-loxP EYFP	Gift from D. Green	N/A
Oligonucleotides		
Human Drp1 sgRNA, knockout: AAATCAGAGAGCTCATTCTT	This paper	N/A
Mouse Dff40 sgRNA, knockout: ACATGGAGCCAAGGACTCGC	This paper	N/A
Human BCL-2 sgRNA, knockin: ATGGCG-CACGCTGGGAGAAC	This paper	N/A
Human BCL-xL sgRNA, knockin: AAAAATGTCTCAGAGCAACC	This paper	N/A
Human MCL-1 sgRNA, knockin: CGGCGGCGACTGGCAATGTT	This paper	N/A
Recombinant DNA		
LZRS	This paper	N/A
LZRS-MFN2	This paper	N/A
LZRS-DRP1	This paper	N/A
pUC Scarlet-BCL-2	This paper	N/A
pUC Scarlet- BCL-xL	This paper	N/A
pUC Scarlet-MCL-1	This paper	N/A
pSpCas9(BB)-2A-Puro	Addgene; Ran et al., 2013	RRID:Addgene_48139
pSpCas9(BB)-2A-Puro human BCL-2	This paper	N/A
pSpCas9(BB)-2A-Puro human BCL-xL	This paper	N/A
pSpCas9(BB)-2A-Puro human MCL-1	This paper	N/A

(*Continued on next page*)

Continued

REAGENT or RESOURCE	SOURCE	IDENTIFIER
LZRS GFP-BAX	Tait et al., 2010	N/A
Omi-GFP	Gift from D. Green	N/A
pBABE iRFP670-Omp25	This paper	N/A
pBABEhygro Myc	This paper	N/A
pBABEpuro HRas	N/A	N/A
LentiCRISPR v2	Sanjana et al., 2014	RRID:Addgene_52961
LentiCRISPR v2 human Drp1	This paper	N/A
LentiCRISPR v2 mouse Dff40	This paper	N/A
gag/pol	Addgene; Reya et al., 2003	RRID:Addgene_14887
pCMV-VSV-G	Addgene, Stewart et al., 2003	RRID:Addgene_8454
psPAX2	Addgene, Trono Lab	RRID:Addgene_12260
Software and algorithms		
ImageJ	Schneider et al., 2012	https://imagej.nih.gov/ij/
FlowJo	BD	RRID:SCR_008520
Zeiss ZEN Black	Zeiss	RRID:SCR_018163
Prism v9.0	GraphPad	RRID:SCR_002798

RESOURCE AVAILABILITY

Lead contact

Further information and requests for resources and reagents should be directed to, and will be fulfilled by, the lead contact, Stephen Tait (stephen.tait@glasgow.ac.uk).

Materials availability

Plasmids and cell lines generated in this study will be made available on request to the lead contact.

Data and code availability

Data reported in this paper will be shared by the lead contact upon request. RNA-seq and mutational count data are available from cBioportal (www.cbioportal.org).

EXPERIMENTAL MODEL AND SUBJECT DETAILS

HeLa and U2OS cells were purchased from ATCC (LGC Standards). 293FT cells were purchased from Thermo Fisher Scientific.

Mfn1/2^{-}/$^{-}$ MEF were provided by David Chan, Caltech and reconstituted with LZRS-MFN2 in our laboratory. *Drp1*$^{fl/fl}$ MEF were provided by Hiromi Sesaki, Johns Hopkins University School of Medicine. MEF Wt and MEF loxP-STOP-loxP EYFP were a kind gift from Douglas Green, St Jude's Research Children's Hospital. All cell lines were cultured in DMEM high-glucose medium supplemented with 10% FCS, 2 mM glutamine, 1 mM sodium pyruvate, penicillin (10,000 units/ml) and streptomycin (10,000 units/ml).

To delete Drp1 from *Drp1*$^{fl/fl}$ MEF, 2 x 10^6 cells were seeded and infected with 200 MOI Ad5CMVCre (Viral Vector Core, University of Iowa) for 8 h, after which the media was replaced. Cells were used for experiments from the following day.

METHOD DETAILS

Generation of Scarlet-BCL-2 knock-in cell lines

We used a modified version of the knock-in strategy described in Stewart-Ornstein and Lahav (2016). Two vectors were used: the first vector comprises 500bp homology arm before and after the start codon of BCL-2, in between which is the Scarlet coding sequence, cloned into pUC-SP. The second vector, pSpCas9(BB)-2A-Puro (Addgene #48139) comprises Cas9 and the sgRNA targeting sequence. The following sgRNA sequences were used

Human BCL-2	5'- ATGGCGCACGCTGGGAGAAC -3'
Human BCL-xL	5- AAAAATGTCTCAGAGCAACC -3'
Human MCL-1	5'- CGGCGGCGACTGGCAATGTT -3'

To generate the knock-in cells, HeLa cells were transfected with 1 μg of homology arm vector and 1 μg of pSpCas9(BB)-2A-Puro with Lipofectamine 2000, according to the manufacturer's instructions. Media was removed 5 h later, and replaced with media containing 1 μM SCR7 for 2 days. Cells were selected with 1 μg/mL puromycin for a further two days before selecting Scarlet positive clones by FACS. Cells which expressed Scarlet signal which co-localised with mitochondria we used for further experiments.

Generation of stable overexpressing cell lines

For retroviral transduction, 293FT cells were transfected with 5 μg of plasmid, together with 1.2 μg gag/pol (Addgene #14887) and 2.4 μg VSVG (Addgene #8454) using Lipofectamine 2000. Media was changed after 6 hours and collected, filtered and used to infect cells 24 and 48 h post-transfection in the presence of 1 μg/ml Polybrene. 24 h following infection, cells were allowed to recover in fresh medium and incubated with selection antibiotic 24 h after. Cells were selected with appropriate antibiotic or FACS sorted to isolate a high-expressing population. Concentrations used for antibiotic selection were 200 μg/ml zeocin (Invivogen) or 1 μg/ml puromycin (Sigma).

For lentiviral transduction, the procedure was the same as for retroviral transduction, except 5 μg plasmid was transfected into 293FT along with 1.86 μg psPAX2 (Addgene #12260) and 1 μg VSVG (Addgene #8454) using Lipofectamine 2000.

Generation of CRISPR knock-out cell lines

Human Drp1 and mouse Dff40 knock-out cell lines were generated by CRISPR-Cas9 gene deletion, using the lentiviral transduction protocol above. The following sequences were cloned into LentiCRISPRv2-puro (Addgene #52961)

Human Drp1:	5'- AAATCAGAGAGCTCATTCTT – 3'
Mouse Dff40:	5'- ACATGGAGCCAAGGACTCGC -3'

Plasmids

LZRS-Drp1 was generated by cloning the Drp1 coding sequence from pcDNA3.1(+) Drp1 (Addgene #34706) into LZRS backbone using Gibson Assembly. pBABE iRFP-Omp25 was cloned by Gibson Assembly using fragments derived from pLJM2 SNAP-Omp25 (Addgene #69599) and pMito-iRFP670 (Addgene #45462). Omi-GFP (in eGFPN2) was a kind gift from Douglas Green, St Jude's Children's Research Hospital.

Western blotting

Cells were collected and lysed in NP-40 lysis buffer (1% NP-40, 1 mM EDTA, 150 mM NaCl, 50 mM Tris-Cl pH 7.4) supplemented with complete protease inhibitor (Roche). Protein concentration of cleared lysates was determined by Bradford assay (Bio-Rad). Equal amounts of protein lysates were subjected to electrophoresis through 10 or 12% SDS-PAGE gels and transferred onto nitrocellulose membranes, which were blocked for 1 h in 5% milk/PBS-Tween at room temperature. Membranes were incubated with primary antibody overnight at 4°C overnight. After washing, membranes were incubated with either goat-anti-rabbit Alexa Fluor 800, goat-anti-mouse Alexa Fluor 680 or goat-anti-rat DyLight 800 for 1 h at room temperature before detection using a Li-Cor Odyssey CLx (Li-Cor).

Flow cytometry

For measuring levels of γH2AX, cells were trypsinised and washed once with PBS and fixed in 4% PFA for 15 minutes at room temperature. After washing once in PBS, cells were resuspended in 300 μL and 700 μL cold ethanol added dropwise while slowly vortexing. Samples were frozen at -20°C overnight. The following day, samples were washed with PBS and blocked in 2% BSA in PBS for 1 h at room temperature and incubated with anti-γH2AX antibody conjugated to Alexa Fluor 647 (Biolegend) for 30 minutes protected from light. Samples were analysed on the BD LSRFortessa flow cytometer (BD Biosciences) using standard protocols.

To measure mitochondrial potential in *Mfn1/2*$^{-/-}$ and *Mfn1/2*$^{-/-}$ + Mfn2 MEF, cells were incubated with 50nM MitoTracker CMXRos (Thermo Fisher Scientific) for 15 mins before collection. Cells were analysed on a Attune NxT flow cytometer (Thermo Fisher Scientific) using standard protocols, and analysed in FlowJo (BD).

PALA assay and Cad genomic amplification

Cells were seeded in triplicate in 6 well plates at a density of 2500 cells per well and cultured in nucleoside-free α-MEM medium supplemented with 10% dialysed FBS. PALA was added at the LD_{50} dose and cells maintained until visible colonies formed. Colonies were fixed and stained in methylene blue (1% methylene blue in 50:50 methanol:water).

To assay Cad genomic amplification, DNA was extracted from PALA resistant colonies, or, in the case of control treated cells where no colonies were viable, DNA was extracted from cells passaged twenty times in DMSO, but not subjected to PALA treatment.

Anchorage-independent growth assay

A 1% base low melting temperature agarose solution (Sigma-Aldrich) was added to 6 well plates and allowed to set. 7,500 cells were suspended in 0.6% agarose in a 1:1: ratio to achieve a final concentration of 0.3% agarose., which was added on top of base agarose. When set, the cell/agarose mix was overlaid with complete DMEM media and colonies counted 14 days later from 15 fields of view per cell line.

qPCR

Genomic DNA was isolated from cells using the GeneJET DNA Extraction Kit (Thermo Fisher Scientific). PCR was performed on a Bio-Rad C1000 Thermal Cycler using the following conditions: 3 min at 95°C, 40 cycles of 20 s at 95°C, 30 s at 57°C, 30 s at 72°C and a final 5 min at 72°C using Brilliant III Ultra-Fast SYBR Green qPCR Master Mix (Agilent Technologies). Relative DNA quantification was analysed by the $2^{-\Delta\Delta Ct}$ method. Primer sequences used are as follows:

Mouse CAD-F	AAGCTCAGATCCTAGTGCTAACG
Mouse CAD-R	CCGTAGTTGCCGATGAGAGG
Mouse 18S-F	ATGGTAGTCGCCGTGCCTAC
Mouse 18S-R	CCGGAATCGAACCCTGATT

Microscopy

Fixed cell imaging

Cells were grown on coverslips and fixed in 4% PFA/PBS for 10 min, followed by permeabilization in 0.2% Triton-X-100/PBS for 15 min. Cells were blocked for 1 h in 2% BSA/PBS and incubated with primary antibodies overnight at 4°C in a humidified chamber. The following day, cells were washed in PBS and secondary antibodies added for 1 h at room temperature, before final wash steps and mounting in Vectashield antifade mounting media.

MOMP assay

Cells were transfected with 250ng CytoGFP and 250ng mito-mCherry for 16 h with either Lipofectamine 2000 or GeneJuice before treatment in combination with 50 nM A/C heterodimizer (Clontech). Minority MOMP was scored based on co-localisation of CytoGFP with mito-mCherry. A minimum of 100 cells were analysed per condition, and we defined minority MOMP as a cell which has 1 or more instances of cytoGFP/mito-mCherry co-localisation.

Airyscan super-resolution imaging

Super-resolution Airyscan images were acquired on a Zeiss LSM 880 with Airyscan microscope (Carl Zeiss). Data were collected using a 63 x 1.4 NA objective for the majority of experiments, although some were acquired using a 40 x 1.3 NA objective. 405nm, 561nm and 640 nm laser lines were used, in addition to a multi-line argon laser (488nm) and images acquired sequentially using the optimal resolution determined by the Zeiss ZEN software. When acquiring z-stacks, the software-recommend slice size was used. Live-cell experiments were performed in an environmental chamber at 37°C and 5% CO_2. Airyscan processing was performed using the Airyscan processing function in the ZEN software, and to maintain clarity some images have been pseudocloured and brightness and contrast altered in FIJI (ImageJ v2.0.0).

Nikon A1R imaging

Confocal images were acquired on a Nikon A1R microscope (Nikon). Data were collected using a 60 x Plan Apo VC Oil DIC N2 objective. 405nm, 561nm, 638nm laser lines were used, in addition to a multi-line argon laser (488nm). Images were acquired sequentially to avoid bleedthrough. For live-cell imaging, cells were imaged in a humidified environmental chamber at 37°C and 5% CO_2. Images were minimally processed in FIJI (ImageJ v2.0.0) to adjust brightness and contrast.

3D rendering and image analysis

Z-stacks acquired on the Zeiss LSM 880 with Airyscan microscope were imported into Imaris (Bitplane, Switzerland). To segment Omi and BCL-2, a surface was created using the Omi-GFP pixel information. Masks were applied to differentiate between BCL-2 inside and outside the Omi surface. From these masks, spots were created from the BCL-2 channel and quantified based on intensity of BCL-2 on mitochondria undergoing minority MOMP.

Fluorescence Loss in Photobleaching

The time lapse images were taken at one frame per second on a Zeiss LSM880 microscope in a 37°C chamber, with a Fluor 40x/1.30 NA oil immersion lens. Cells were in HEPES containing media. Fluorescence was excited using the 488nm line of an argon ion laser at 5.4% power through the AOTF and the emitted signal was captured with an Airyscan detector in Resolution vs. Signal mode using a combination of LP500 and BP485-550 filters with a zoom of 3.6. A letterbox bleach region covering approximately a third of the cell area was achieved using combination of 405 and 488nm wavelengths of light at 100% laser power and 100 iterations which took approximately 14 seconds.

Digitionin permeabilisation

Prior to digitonin permeabilisation, cells were incubated in FluoroBrite DMEM without FBS. To permeabilise the plasma membrane, 20 μM digitonin (Sigma) was added and cells imaged immediately.

Mitochondrial analysis

Cells stained with TOM20 and imaged on the Zeiss LSM 880 with Airyscan. These images were analysed by dividing mitochondrial length by width (mitochondria aspect ratio) in ImageJ. Heterogeneity of BCL-2 localisation was measured by calculating the standard deviation of Scarlet and mitochondrial signals in mitochondrial regions in FIJI (ImageJ v2.0.0).

Live-cell viability assays

Cell viability was assayed using either an IncuCyte ZOOM or IncuCyte S3 imaging system (Sartorius). Cells were seeded overnight and drugged in the presence of 30 nM SYTOX Green (Thermo Fisher Scientific), which is a non-cell-permeable nuclear stain. Data were analysed in the IncuCyte software, and where different cell lines are compared the data are normalised to starting confluency.

Bioinformatic analysis

Relationship between DRP1 (*DNM1L*) expression and mutational count were investigated in TCGA PanCancer Atlas studies through cBioportal (Cerami et al., 2012; Gao et al., 2013). Studies with greater than 100 samples were analysed and samples divided into quartiles of DNM1L: mRNA expression z-scores relative to diploid samples (RNA Seq V2 RSEM). Of these, a significant association between increased mutational count in *DNM1L* mRNA highest quartile versus *DNM1L* mRNA lowest quartile was found in 2 out of 22 studies with the inverse relationship not observed in any cancer type. Mutation count in *DNM1L* quartiles was viewed in the Clinical Tab, statistical analysis of mutation count was performed by cBioportal, Wilcoxon test, q-value <0.05 was considered significant. As the relationship between DNM1L and mutational count was highly significant in Invasive Breast Carcinoma and Non-Small Cell Lung Cancer, we used these studies for further interrogation with cases of Lung adenocarcinoma selected from Non-Small Cell Lung Cancer dataset (not Lung squamous cell carcinoma). Data were downloaded from cBioportal and mutational count in *DNM1L* mRNA highest quartile versus *DNM1L* mRNA lowest quartile (mRNA expression z-scores relative to diploid samples (RNA Seq V2 RSEM)) plotted in GraphPad Prism Version 9.0.0 and statistical significance between groups calculated by Mann-Whitney test. Data points represent individual patient samples, bar is mean (SD). *DNM1L* quartiles each contain 128 samples (Lung Adenocarcinoma TCGA PanCancer Atlas dataset) or 271 samples (Breast Invasive Carcinoma TCGA PanCancer Atlas dataset). Differentially expressed proteins in DNM1L highest versus lowest quartiles were also determined in cBioportal (measured by reverse-phase protein array, Z-scores) where significant differences are determined by Student's t-test (*p* value) and Benjamini-Hochberg procedure (*q* value). Pathway analysis was performed using gene names of proteins identified with significantly higher expression in DNM1L high versus DNM1L low quartiles (excluding phospho-specific proteins, see lists in Table S1) in GO Biological Process 2018 through Enrichr (Chen et al., 2013; Kuleshov et al., 2016).

Relationship between DRP1 (DNM1L) expression and patient survival were investigated in TCGA PanCancer Atlas studies through cBioportal (Cerami et al., 2012; Gao et al., 2013). Lung Adenocarcinoma TCGA PanCancer Atlas and Breast Invasive Carcinoma TCGA PanCancer Atlas datasets were downloaded from cBioportal, samples divided into quartiles of DNM1L: mRNA expression (z-scores relative to normal samples log RNA Seq V2 RSEM), with survival of highest and lowest quartiles plotted in GraphPad Prism Version 9.0.0 and statistical significance between groups calculated by Log-rank (Mantel-Cox) test.

Developmental Cell, Volume *57*

Supplemental information

Mitochondrial dynamics regulate genome stability via control of caspase-dependent DNA damage

Kai Cao, Joel S. Riley, Rosalie Heilig, Alfredo E. Montes-Gómez, Esmee Vringer, Kevin Berthenet, Catherine Cloix, Yassmin Elmasry, David G. Spiller, Gabriel Ichim, Kirsteen J. Campbell, Andrew P. Gilmore, and Stephen W.G. Tait

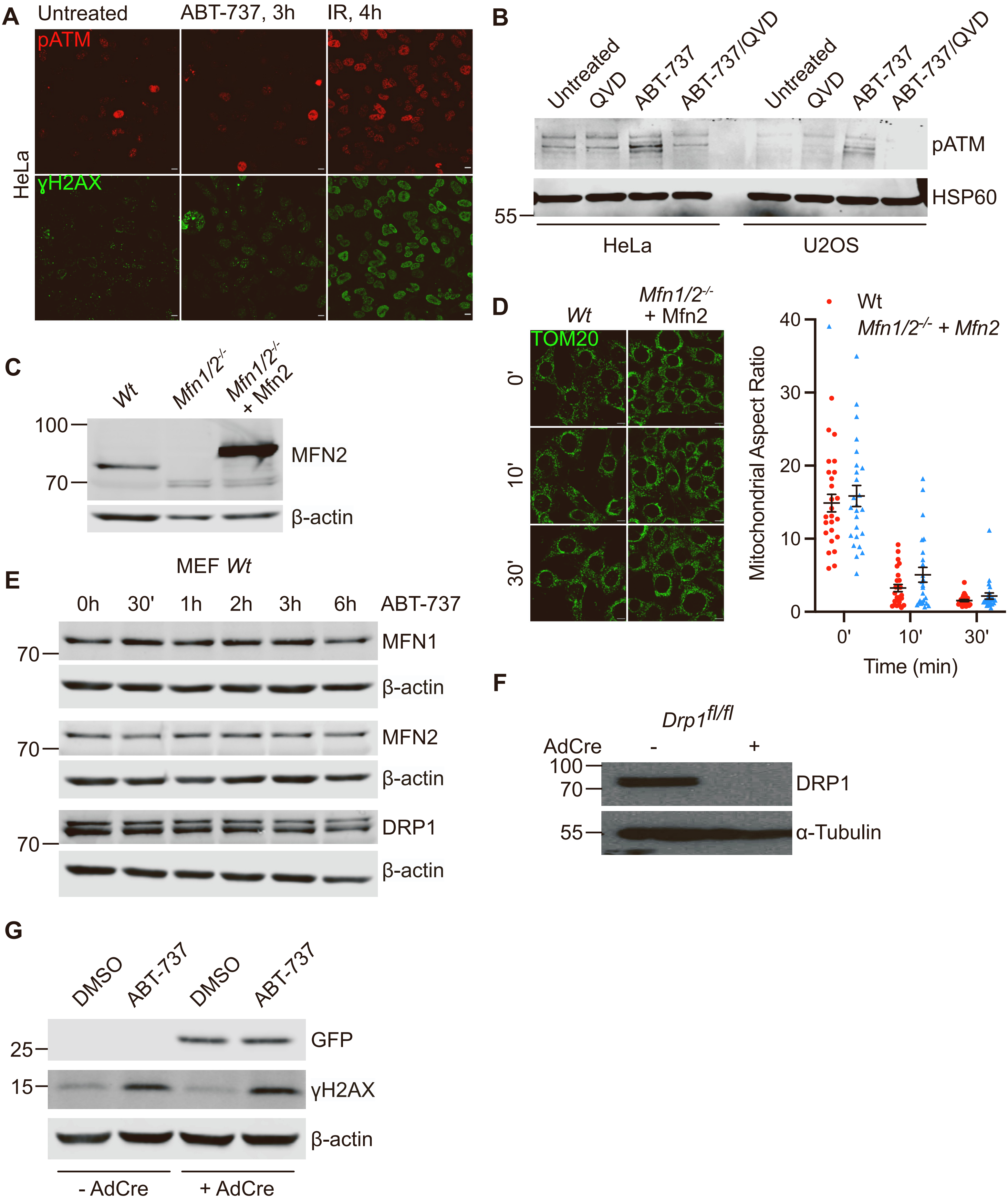
A
Untreated
ABT-737, 3h
IR, 4h
HeLa
pATM
γH2AX
B
Untreated
QVD
ABT-737
ABT-737/QVD
Untreated
QVD
ABT-737
ABT-737/QVD
pATM
HSP60
55
HeLa
U2OS
C
Wt
Mfn1/2-/-
Mfn1/2-/- + Mfn2
100
70
MFN2
β-actin
D
Wt
Mfn1/2-/- + Mfn2
TOM20
0'
10'
30'
Wt
Mfn1/2-/- + Mfn2
Mitochondrial Aspect Ratio
40
30
20
10
0
0'
10'
30'
Time (min)
E
MEF Wt
0h
30'
1h
2h
3h
6h
ABT-737
MFN1
70
β-actin
MFN2
70
β-actin
DRP1
70
β-actin
F
Drp1fl/fl
AdCre
-
+
100
70
DRP1
55
α-Tubulin
G
DMSO
ABT-737
DMSO
ABT-737
GFP
25
15
γH2AX
β-actin
- AdCre
+ AdCre

Figure S1. Mitochondrial dynamics regulate DNA damage. Related to Figure 1

A) Airyscan images of HeLa cells treated with 10 µM ABT-737 for 3 h, or 20 Gy irradiation for 4 h. Cells were immunostained with anti-pATM (red) or anti-ɣH2AX (green) antibody. Images representative of 3 independent experiments. Scale bar = 10 µm

B) Western blot of HeLa and U2OS cells treated with 10 µM QVD, 10 µM ABT-737 or a combination of both for 3 h. Blots were immunoblotted for pATM and HSP60 and a representative of 2 independent experiments.

C) Western blot of *Wt*, $Mfn1/2^{-/-}$ and $Mfn1/2^{-/-}$ + Mfn2 MEF immunoblotted for MFN2 and β-actin. Representative of 2 independent experiments.

D) Airyscan images of *Wt* or $Mfn1/2^{-/-}$ + Mfn2 MEF treated with 10 µM CCCP for the indicated times and immunostained with anti-TOM20 antibody. Mitochondrial aspect ratio data is from 3 independent experiments. Data are expressed as mean ± SEM.

E) Western blot of *Wt* MEF treated with 10 µM ABT-737 for 3 h, and immunoblotted for MFN1, MFN2, DRP1 and β-actin. Data are representative of 3 independent experiments.

F) Western blot of $Drp1^{fl/fl}$ MEF with and without adenoviral Cre infection. Lysates immunoblotted for DRP1 and α-tubulin.

G) Western blot of loxP-STOP-loxP MEF infected or not with adenoviral Cre to induce YFP expression. Cells were treated with 10 µM ABT-737 for 3 h and YFP expression analysed by GFP immunoblotting, DNA damage by ɣH2AX and β-actin for loading control. Data is representative of 3 independent experiments.

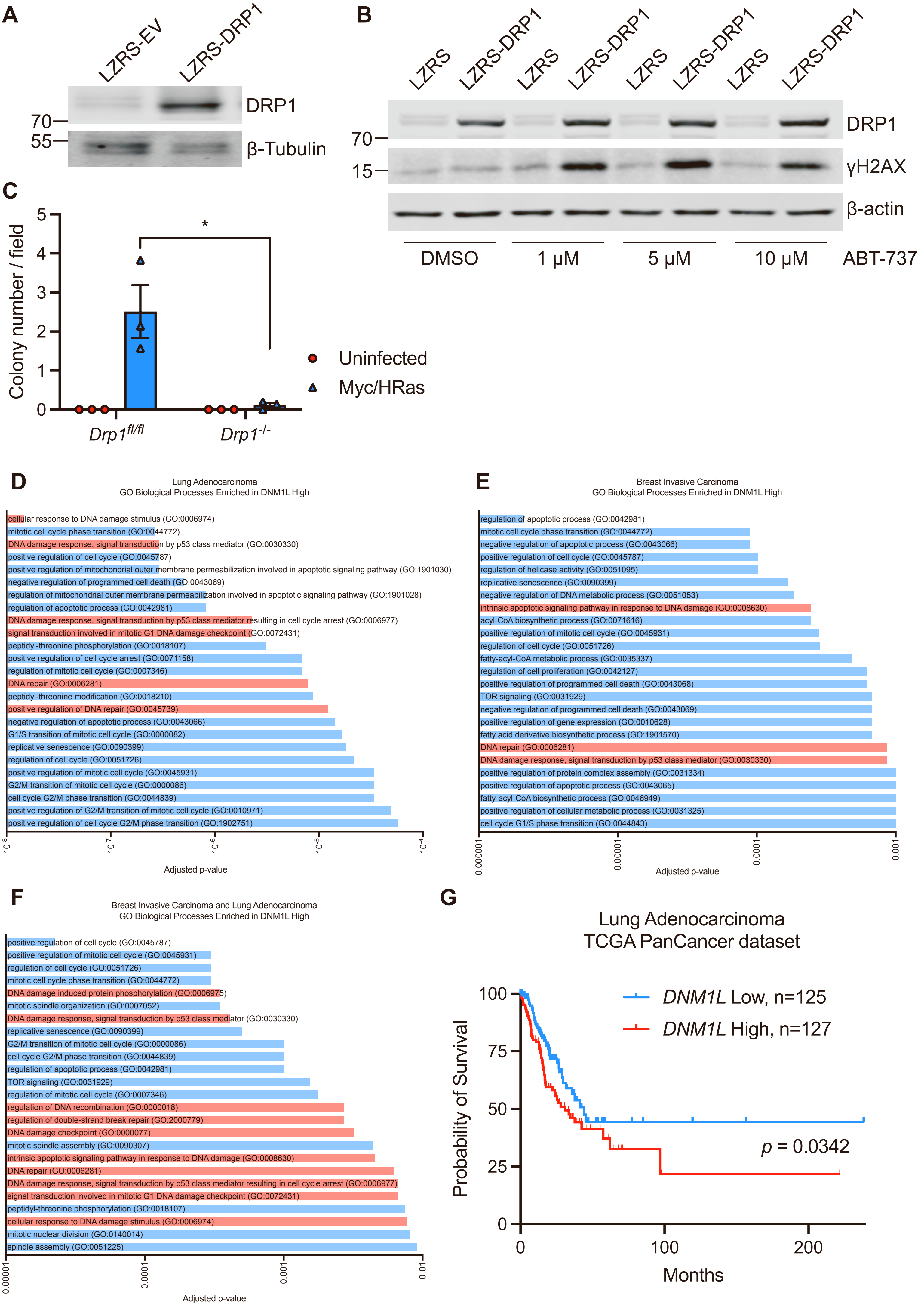

A
LZRS-EV
LZRS-DRP1
DRP1
70
55
β-Tubulin
B
LZRS
LZRS-DRP1
LZRS
LZRS-DRP1
LZRS
LZRS-DRP1
LZRS
LZRS-DRP1
DRP1
70
15
γH2AX
β-actin
DMSO
1 μM
5 μM
10 μM
ABT-737
C
Colony number / field
*
Uninfected
Myc/HRas
Drp1fl/fl
Drp1-/-
D
Lung Adenocarcinoma
GO Biological Processes Enriched in DNM1L High
cellular response to DNA damage stimulus (GO:0006974)
mitotic cell cycle phase transition (GO:0044772)
DNA damage response, signal transduction by p53 class mediator (GO:0030330)
positive regulation of cell cycle (GO:0045787)
positive regulation of mitochondrial outer membrane permeabilization involved in apoptotic signaling pathway (GO:1901030)
negative regulation of programmed cell death (GO:0043069)
regulation of mitochondrial outer membrane permeabilization involved in apoptotic signaling pathway (GO:1901028)
regulation of apoptotic process (GO:0042981)
DNA damage response, signal transduction by p53 class mediator resulting in cell cycle arrest (GO:0006977)
signal transduction involved in mitotic G1 DNA damage checkpoint (GO:0072431)
peptidyl-threonine phosphorylation (GO:0018107)
positive regulation of cell cycle arrest (GO:0071158)
regulation of mitotic cell cycle (GO:0007346)
DNA repair (GO:0006281)
peptidyl-threonine modification (GO:0018210)
positive regulation of DNA repair (GO:0045739)
negative regulation of apoptotic process (GO:0043066)
G1/S transition of mitotic cell cycle (GO:0000082)
replicative senescence (GO:0090399)
regulation of cell cycle (GO:0051726)
positive regulation of mitotic cell cycle (GO:0045931)
G2/M transition of mitotic cell cycle (GO:0000086)
cell cycle G2/M phase transition (GO:0044839)
positive regulation of G2/M transition of mitotic cell cycle (GO:0010971)
positive regulation of cell cycle G2/M phase transition (GO:1902751)
10-8
10-7
10-6
10-5
10-4
Adjusted p-value
E
Breast Invasive Carcinoma
GO Biological Processes Enriched in DNM1L High
regulation of apoptotic process (GO:0042981)
mitotic cell cycle phase transition (GO:0044772)
negative regulation of apoptotic process (GO:0043066)
positive regulation of cell cycle (GO:0045787)
regulation of helicase activity (GO:0051095)
replicative senescence (GO:0090399)
negative regulation of DNA metabolic process (GO:0051053)
intrinsic apoptotic signaling pathway in response to DNA damage (GO:0008630)
acyl-CoA biosynthetic process (GO:0071616)
positive regulation of mitotic cell cycle (GO:0045931)
regulation of cell cycle (GO:0051726)
fatty-acyl-CoA metabolic process (GO:0035337)
regulation of cell proliferation (GO:0042127)
positive regulation of programmed cell death (GO:0043068)
TOR signaling (GO:0031929)
negative regulation of programmed cell death (GO:0043069)
positive regulation of gene expression (GO:0010628)
fatty acid derivative biosynthetic process (GO:1901570)
DNA repair (GO:0006281)
DNA damage response, signal transduction by p53 class mediator (GO:0030330)
positive regulation of protein complex assembly (GO:0031334)
positive regulation of apoptotic process (GO:0043065)
fatty-acyl-CoA biosynthetic process (GO:0046949)
positive regulation of cellular metabolic process (GO:0031325)
cell cycle G1/S phase transition (GO:0044843)
0.000001
0.00001
0.0001
0.001
Adjusted p-value
F
Breast Invasive Carcinoma and Lung Adenocarcinoma
GO Biological Processes Enriched in DNM1L High
positive regulation of cell cycle (GO:0045787)
positive regulation of mitotic cell cycle (GO:0045931)
regulation of cell cycle (GO:0051726)
mitotic cell cycle phase transition (GO:0044772)
DNA damage induced protein phosphorylation (GO:0006975)
mitotic spindle organization (GO:0007052)
DNA damage response, signal transduction by p53 class mediator (GO:0030330)
replicative senescence (GO:0090399)
G2/M transition of mitotic cell cycle (GO:0000086)
cell cycle G2/M phase transition (GO:0044839)
regulation of apoptotic process (GO:0042981)
TOR signaling (GO:0031929)
regulation of mitotic cell cycle (GO:0007346)
regulation of DNA recombination (GO:0000018)
regulation of double-strand break repair (GO:2000779)
DNA damage checkpoint (GO:0000077)
mitotic spindle assembly (GO:0090307)
intrinsic apoptotic signaling pathway in response to DNA damage (GO:0008630)
DNA repair (GO:0006281)
DNA damage response, signal transduction by p53 class mediator resulting in cell cycle arrest (GO:0006977)
signal transduction involved in mitotic G1 DNA damage checkpoint (GO:0072431)
peptidyl-threonine phosphorylation (GO:0018107)
cellular response to DNA damage stimulus (GO:0006974)
mitotic nuclear division (GO:0140014)
spindle assembly (GO:0051225)
0.00001
0.0001
0.001
0.01
Adjusted p-value
G
Lung Adenocarcinoma
TCGA PanCancer dataset
DNM1L Low, n=125
DNM1L High, n=127
Probability of Survival
p = 0.0342
Months

Figure S2. Mitochondrial fission regulates DNA damage, transformation and cancer survival. Related to Figure 2

A) Western blot of MEF expressing LZRS empty control, or LZRS-DRP1. Lysates immunoblotted for DRP1 and β-tubulin.

B) MEF stably overexpressing LZRS-DRP1 or LZRS empty vector were treated with the indicated dose of ABT-737 for 3 h and lysates immunoblotted for DRP1, ɣH2AX and β-actin. Data are representative of 3 independent experiments.

C) *Drp1*$^{fl/fl}$ and *Drp1*$^{-/-}$ MEF infected with viruses encoding Myc and HRas were cultured in soft agar and colony number quantified. Data are from 3 independent experiments, expressed as mean ± SEM and analysed using student's t-test.

D) Gene ontology (GO) biological processes significantly up-regulated in DNM1L high expressing lung adenocarcinoma samples. Bars in red represent GO biological processes related to DNA damage and are expressed as adjusted p-values.

E) Gene ontology (GO) biological processes significantly up-regulated in DNM1L high expressing breast invasive carcinoma samples. Bars in red represent GO biological processes related to DNA damage and are expressed as adjusted p-values.

F) Gene ontology (GO) biological processes significantly up-regulated in DNM1L high expressing lung adenocarcinoma and breast invasive carcinoma samples. Bars in red represent GO biological processes related to DNA damage and are expressed as adjusted p-values.

G) Survival of lung adenocarcinoma patients. DNM1L Low refers to those patients in the lowest quartile of DNM1L mRNA expression, and DNM1L High refers to patients in the highest quartile. Significance is calculated by log-rank (Mantel-Cox) test.

Statistics: * $p \leq 0.05$, ** $p \leq 0.01$, *** $p \leq 0.001$

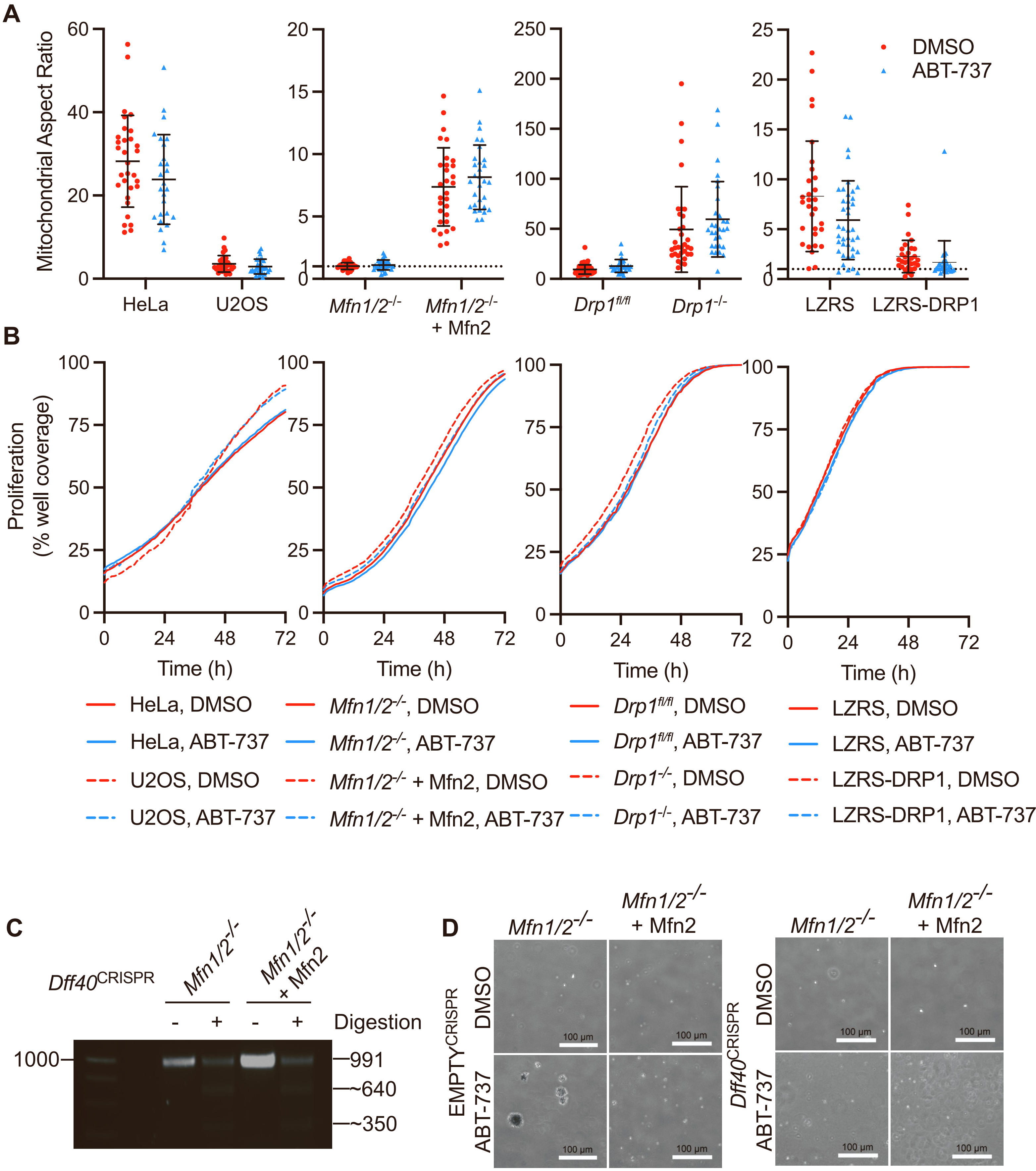
A
Mitochondrial Aspect Ratio
HeLa
U2OS
Mfn1/2-/-
Mfn1/2-/- + Mfn2
Drp1fl/fl
Drp1-/-
LZRS
LZRS-DRP1
DMSO
ABT-737
B
Proliferation (% well coverage)
Time (h)
HeLa, DMSO
HeLa, ABT-737
U2OS, DMSO
U2OS, ABT-737
Mfn1/2-/-, DMSO
Mfn1/2-/-, ABT-737
Mfn1/2-/- + Mfn2, DMSO
Mfn1/2-/- + Mfn2, ABT-737
Drp1fl/fl, DMSO
Drp1fl/fl, ABT-737
Drp1-/-, DMSO
Drp1-/-, ABT-737
LZRS, DMSO
LZRS, ABT-737
LZRS-DRP1, DMSO
LZRS-DRP1, ABT-737
C
Dff40CRISPR
Mfn1/2-/-
Mfn1/2-/- + Mfn2
Digestion
1000
991
~640
~350
D
EMPTYCRISPR
Dff40CRISPR
DMSO
ABT-737
100 µm

Figure S3. Impact of mitochondrial fusion and fission machinery modulation on mitochondrial morphology, growth and CAD-dependent transformation. Related to Figure 4

A) Mitochondria aspect ratio of indicated cell lines treated with or without 10 μM ABT-737 for 3 h. Cells were immunostained for TOM20 and imaged using Airyscan microscopy. Mitochondrial aspect ratio was calculated from 3 independent experiments and expressed as mean (SD).
B) Proliferation of indicated cell lines treated with or without 10 μM ABT-737 for 3h, after which time the media was changed to media not containing ABT-737. Cells were imaged for 72 h.
C) T7 endonuclease I mismatch assay to assay for CAD/*Dff40* deletion.
D) Representative images of anchorage-independent growth of *Mfn1/2*$^{-/-}$ and *Mfn1*$^{-/-}$ MEF with and without CRISPR-Cas9-mediated *Dff40* deletion. Cells were passaged twenty times in 10 μM ABT-737. Scale bar = 100 μm. Data are quantified in **Figure 2K**.

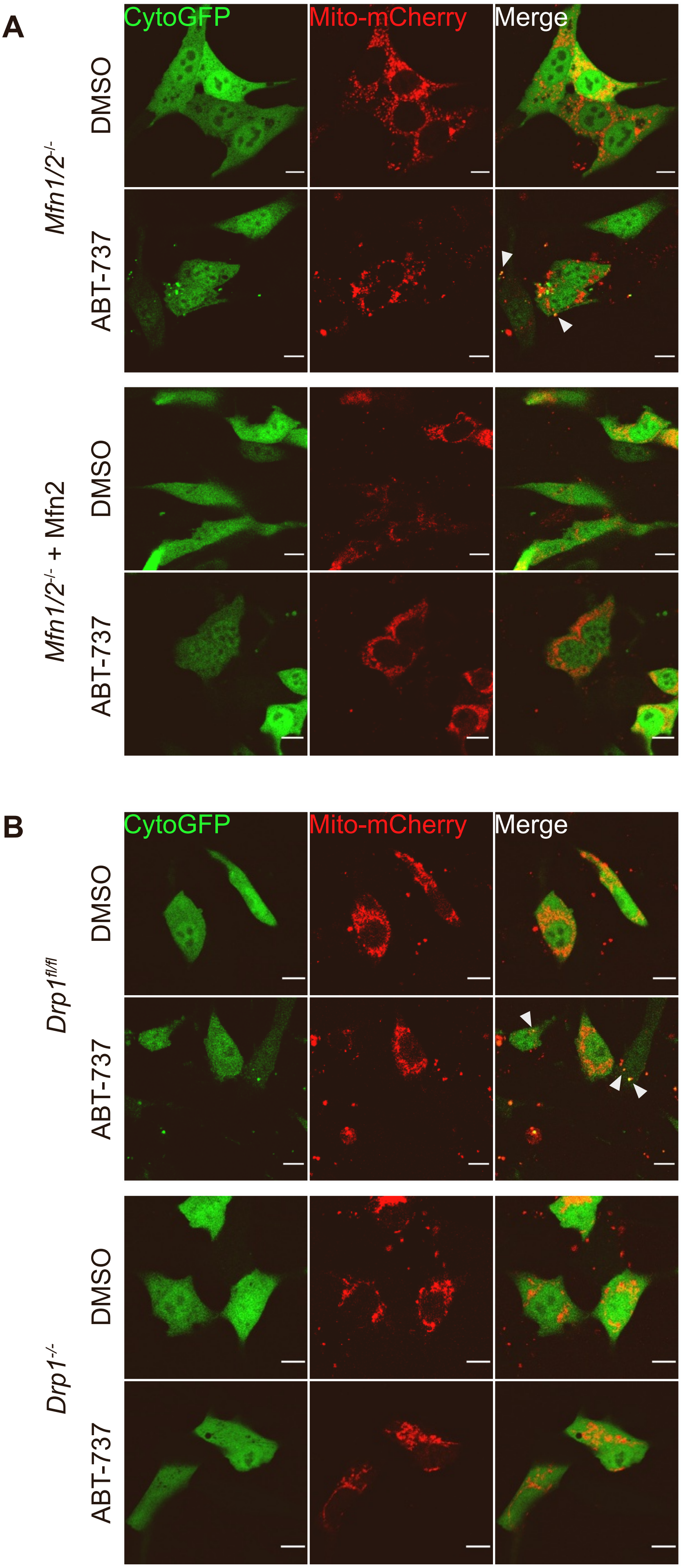

A
CytoGFP
Mito-mCherry
Merge
Mfn1/2-/-
DMSO
ABT-737
Mfn1/2-/- + Mfn2
DMSO
ABT-737
B
CytoGFP
Mito-mCherry
Merge
Drp1fl/fl
DMSO
ABT-737
Drp1-/-
DMSO
ABT-737

Figure S4. Visualisation of minority MOMP in cells with modulated mitochondrial dynamics. Related to Figure 5.

A) Confocal images of *Mfn1/2*$^{-/-}$ and *Mfn1/2*$^{-/-}$ + Mfn2 cells transiently expressing cytoGFP and mito-mCherry. Cells were treated with dimeriser and treated or not with 10 μM ABT-737 for 3 h before fixation, imaging and quantification of minority MOMP. Images are quantified in **Figure 3E**.

B) Confocal images of *Drp1*$^{fl/fl}$ and *Drp1*$^{-/-}$ cells transiently expressing cytoGFP and mito-mCherry. Cells were treated with dimeriser and treated or not with 10 μM ABT-737 for 3 h before fixation, imaging and quantification of minority MOMP. Images are quantified in **Figure 3F**.

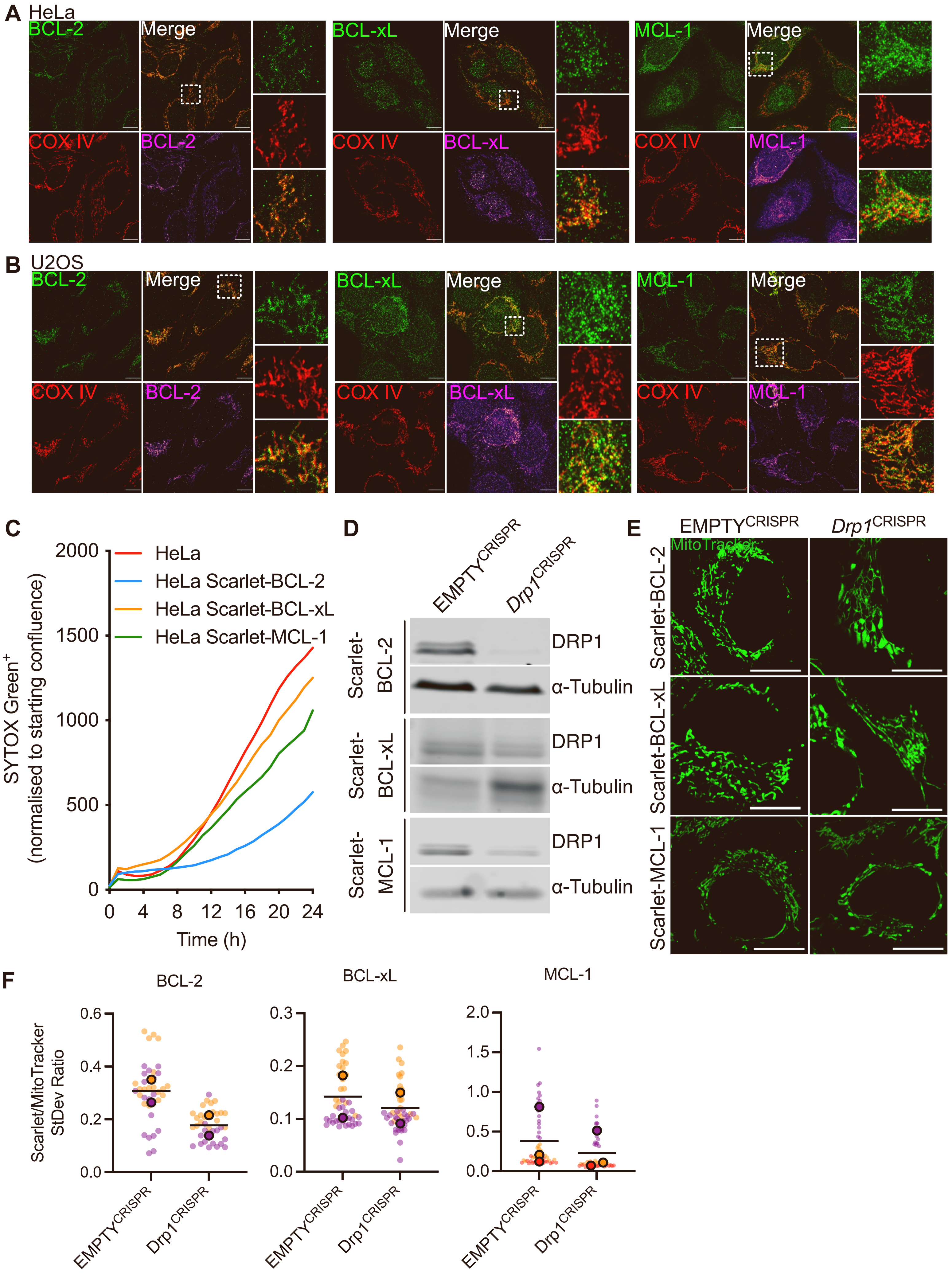
A HeLa
BCL-2
Merge
COX IV
BCL-2
BCL-xL
Merge
COX IV
BCL-xL
MCL-1
Merge
COX IV
MCL-1
B U2OS
BCL-2
Merge
COX IV
BCL-2
BCL-xL
Merge
COX IV
BCL-xL
MCL-1
Merge
COX IV
MCL-1
C
HeLa
HeLa Scarlet-BCL-2
HeLa Scarlet-BCL-xL
HeLa Scarlet-MCL-1
SYTOX Green+ (normalised to starting confluence)
Time (h)
D
EMPTYCRISPR
Drp1CRISPR
Scarlet-BCL-2
Scarlet-BCL-xL
Scarlet-MCL-1
DRP1
α-Tubulin
E
EMPTYCRISPR
Drp1CRISPR
MitoTracker
Scarlet-BCL-2
Scarlet-BCL-xL
Scarlet-MCL-1
F
BCL-2
BCL-xL
MCL-1
Scarlet/MitoTracker StDev Ratio
EMPTYCRISPR
Drp1CRISPR

Figure S5. Characterisation of BCL-2 family knockin cells. Related to Figure 6.

A) Airyscan images of HeLa cells immunostained for BCL-2, BCL-xL or MCL-1 (green) in combination with COX IV (red). Also shown are BCL-2, BCL-xL or MCL-1 images with a magenta LUT applied to visualise heterogeneity. Scale bar = 10 μm. Data are representative of 3 independent experiments.

B) Airyscan images of U2OS cells immunostained for BCL-2, BCL-xL or MCL-1 (green) in combination with COX IV (red). Also shown are BCL-2, BCL-xL or MCL-1 images with a magenta LUT applied to visualise heterogeneity. Scale bar = 10 μm. Data are representative of 3 independent experiments.

C) Live-cell IncuCyte analysis of HeLa and HeLa Scarlet-MCL-1 knockin cells treated with 10 μM ABT-737 and 2 μM S63845. Cells were incubated with SYTOX Green and SYTOX Green positivity was measured over time and normalised to starting confluency.

D) Western blots of HeLa Scarlet-BCL-2, BCL-xL and MCL-1 knockin cells with CRISPR-Cas9-mediated DRP1 deletion. Lysates immunoblotted for DRP1 and α-tubulin as a loading control.

E) Representative Airyscan images of mitochondrial structure in HeLa Scarlet-BCL-2, BCL-xL and MCL-1 knockin cells with CRISPR-Cas9-mediated DRP1 deletion. Scale bar = 10 μm. Data are representative of at least 2 independent experiments.

F) Quantification of standard deviation of Scarlet to MitoTracker signal in HeLa Scarlet BCL-2, BCL-xL and MCL-1 cells, with or without CRISPR-Cas9-mediated DRP1 deletion. Data are expressed as borderless points for individual cells and points with borders represent summary data for n = 2 biological replicates for BCL-2 and BCL-xL, and n = 3 for MCL-1. The line represents the mean.

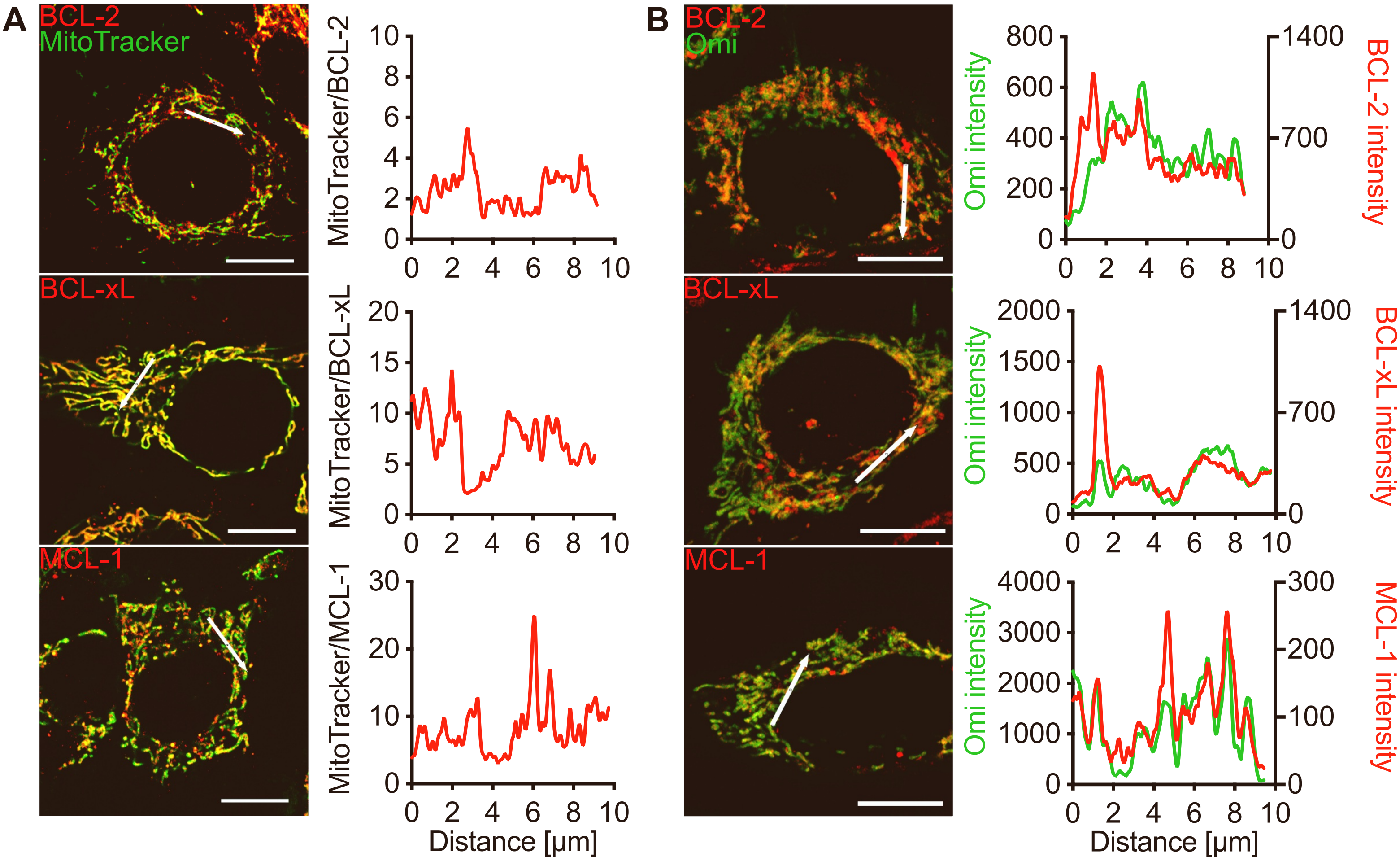

A
BCL-2
MitoTracker
MitoTracker/BCL-2
BCL-xL
MitoTracker/BCL-xL
MCL-1
MitoTracker/MCL-1
Distance [µm]
B
BCL-2
Omi
Omi intensity
BCL-2 intensity
BCL-xL
BCL-xL intensity
MCL-1
MCL-1 intensity
Distance [µm]

Figure S6. Mitochondrial localisation of BCL2-family knockins. Related to Figure 5.

A) Airyscan images of HeLa Scarlet-BCL-2, BCL-xL and MCL-1 knockin cells and MitoTracker. Line scans show ratio of MitoTracker to Scarlet-BCL-2. Scale bar = 10 µm. Data representative of 3 independent experiments.

B) Airyscan images of HeLa Scarlet-BCL-2, BCL-xL and MCL-1 knockin cells and Omi-GFP. Line scans show ratio of Omi-GFP intensity (green) and Scarlet-BCL-2 intensity (red). Scale bar = 10 µm. Data representative of 3 independent experiments.

A

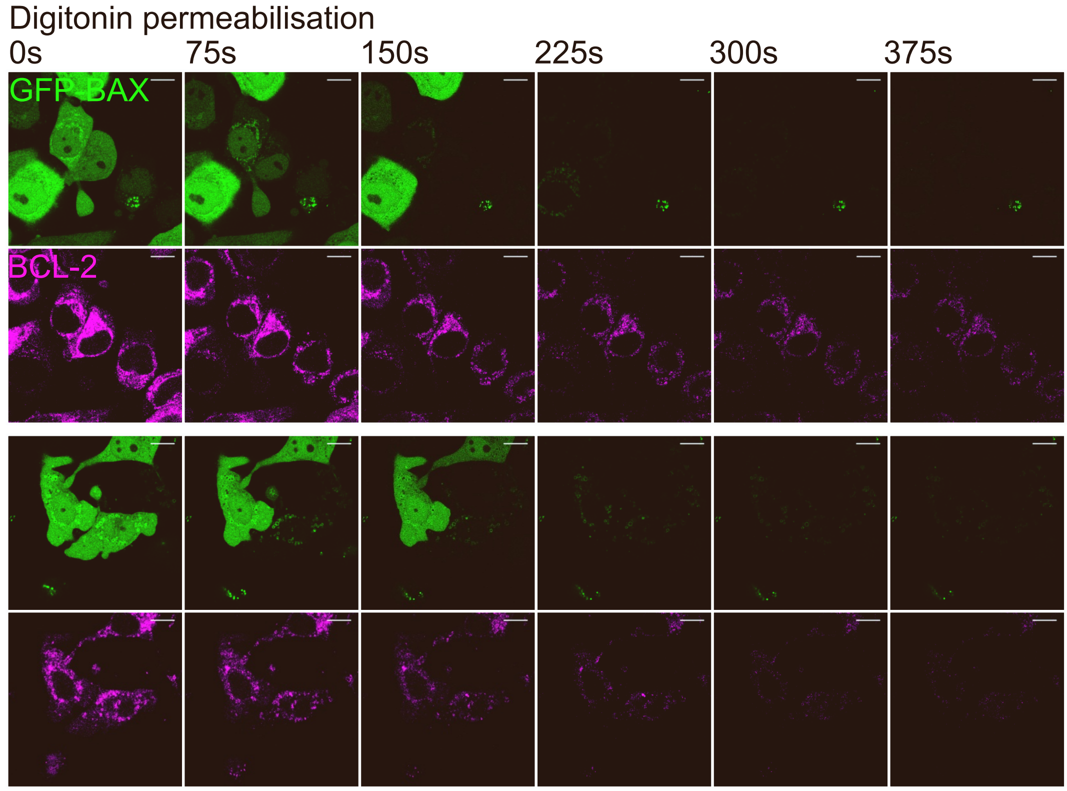

Digitonin permeabilisation
0s
75s
150s
225s
300s
375s
GFP-BAX
BCL-2
Untreated
CCCP

B

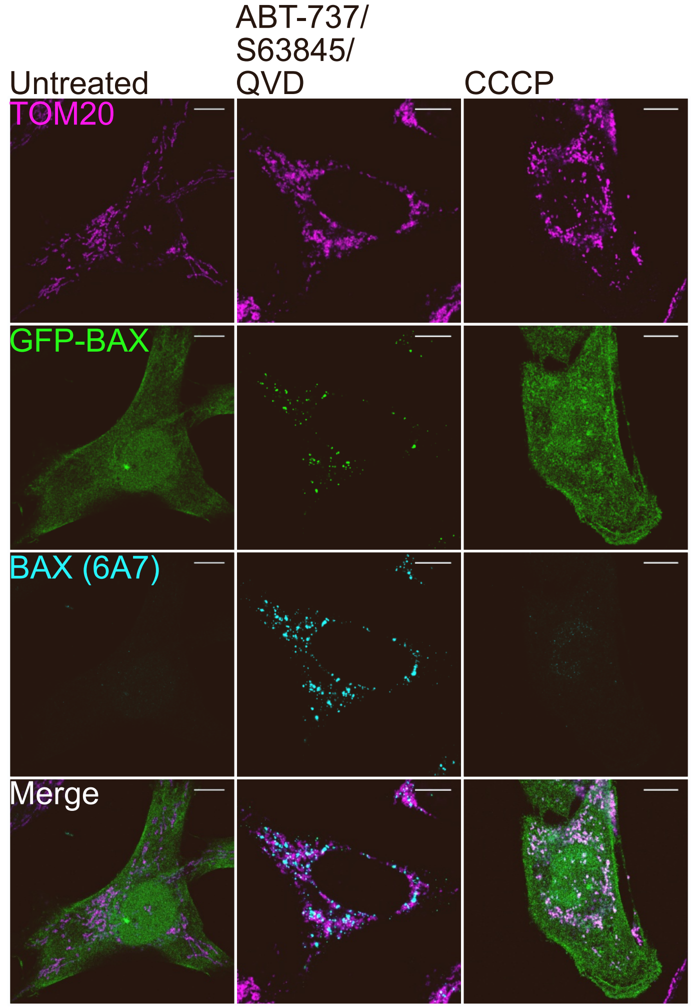

Untreated
ABT-737/
S63845/
QVD
CCCP
TOM20
GFP-BAX
BAX (6A7)
Merge

Figure S7. Mitochondrial dysfunction causes BAX accumulation on the mitochondrial outer membrane, but not BAX pore formation. Related to Figure 6.

A) Images of HeLa Scarlet-BCL-2 knockin (magenta) cells stably overexpressing GFP-BAX (green) treated 10 μM CCCP for 30 min before digitonin permeabilisation. Scale bar = 10 μm. See also **Videos S4, S5**.

B) Airyscan images of fixed HeLa cells stably overexpressing GFP-BAX (green) and treated with 10 μM CCCP or 10 μM ABT-737, 2 μM S63845 and 10 μM QVD for 3 h. Cells were immunostained with anti-TOM20 (magenta) and anti-BAX 6A7 (cyan). Scale bar = 10 μm. Images representative of 3 independent experiments.
